# Physical Intimacy of Breast Cancer Cells with Mesenchymal Stem Cells Elicits Trastuzumab Resistance through Src Activation

**DOI:** 10.1038/srep13744

**Published:** 2015-09-08

**Authors:** Amita Daverey, Allison P. Drain, Srivatsan Kidambi

**Affiliations:** 1 Department of Chemical and Biomolecular Engineering, University of Nebraska-Lincoln, NE, 68588; 2 Nebraska Center for Materials and Nanoscience, University of Nebraska-Lincoln, NE, 68588; 3 Mary and Dick Holland Regenerative Medicine Program, University of Nebraska Medical Center, NE, 68198.

## Abstract

The development of resistance to trastuzumab is a major obstacle for lasting effective treatment of patients with ErbB2-overexpressing tumors. Here, we demonstrate that the physical contact of breast cancer cells with mesenchymal stem cells (MSCs) is a potential modulator of trastuzumab response by activation of nonreceptor tyrosine kinase c-Src and down regulation of phosphatase and tensin homolog (PTEN). Using an *in vitro* patterned breast cancer/MSC co-culture model, we find that the presence of MSCs results in Src activation that is missing in cancer cells monoculture, transwell co-culture, and cells treated with MSCs conditioned media. Interestingly, the co-culture model also results in PTEN loss and activation of PI3K/AKT pathway that has been demonstrated as fundamental proliferative and survival pathways in clinical settings. To our knowledge, this is the *first report* that showed PTEN loss without the use of chemical inhibitors, matrix stiffness, or silencing RNAs. In addition, breast cancer cells in co-culture with MSCs conferred trastuzumab resistance *in vitro* as observed in the lack of inhibition of proliferative and migrative properties of the cancer cells. Our findings show that MSCs are potent mediators of resistance to trastuzumab and might reveal targets to enhance trastuzumab efficacy in patients.

Human epidermal growth factor receptor-2 (HER-2 or ErbB2) gene is overexpressed in approximately 20–25% of human breast cancers and is associated with poor clinical prognosis and survival^1^,^2^. Treatment with trastuzumab, a humanized antibody that targets HER-2, has dramatically altered the course of HER-2 positive breast cancer patients. However, majority of the patients do not respond to initial treatment or develop resistance after continuous treatment of the drug^3^,^4^. Two major trastuzumab resistance mechanisms have been proposed: (i) *de novo* resistance due to genetic alterations of receptor tyrosine kinases (RTKs) and their downstream signaling targets (such as phosphatase and tensin homolog (PTEN) loss and activation of the phosphoinositide 3-kinase (PI3K)); and (ii) acquired resistance primarily due to the acquisition of alternative RTK signaling activation that compensate for HER-2 inhibition after trastuzumab treatment^3^,^4^,^5^. Recent studies have highlighted a new resistance mechanism implicating nonreceptor tyrosine kinase c-Src as a key modulator of trastuzumab response and a common node downstream of multiple trastuzumab- resistance pathways^6^,^7^,^8^. Increased activation of Src has been observed in both acquired and *de novo* trastuzumab-resistant cells and this activation regulates the loss of PTEN, thus promoting drug resistance. Moreover, *in vitro* and *in vivo* experimental results strongly indicate an important role of Src in the development and progression of breast cancer as well as a viable therapeutic option^6^,^7^,^8^. Despite these promising experimental data, the underlying molecular mechanisms of what might activate Src leading to trastuzumab resistance remains unclear.

Tumor microenvironment has garnered the spotlight in recent years for its important role in tumor progression and drug resistance^9^,^10^,^11^. Tumors actively modulate their microenvironment by recruiting lymphocytes and macrophages^12^; vascular endothelial cells; and tumor-associated stromal cells such as tumor-associated fibroblasts (TAFs) and mesenchymal stem cells (MSCs)^13^,^14^. MSCs, in addition to other cells in the tumor microenvironment, have been identified as an important population of cells that modulate tumor progression and drug sensitivity^15^,^16^,^17^. Recent reports have demonstrated that MSCs are recruited in large numbers to the stroma of developing tumors^18^,^19^. Furthermore, MSCs integrate into tumor-associated stroma, and exhibit multiple regulatory functions in the tumor microenvironment^13^,^20^,^21^. The bidirectional paracrine signaling between MSCs and breast cancer cells are found to stimulate tumor growth, enhance angiogenesis, and promote metastasis formation through the release of a large spectrum of growth factors and cytokines^22^,^23^,^24^. MSCs also promote tumor cell migration, an epithelial-to-mesenchymal transition (EMT), and increase chemoresistance in breast cancer cells^15^,^16^,^17^. Growth patterns of cancer cells in co-culture change from a clustered to a single cell distribution, and these morphological alterations have been related to a significant down regulation of cell adhesion molecules E-cadherin and epithelial specific antigen (ESA)^25^. MSCs are also believed to modulate the response to drugs including trastuzumab by either direct cell-cell interactions with tumor cells, or by the local release of soluble factors such as interleukin-6, promoting survival and tumor growth^15^,^26^,^27^,^28^.

The growth- and metastasis-promoting effects of MSCs have been well documented^22^,^23^,^24^, but a possible role in drug resistance including activation of Src and downstream pathways has been only partially explored, and has been difficult to obtain. This is due, in part, to the complexity of recreating and isolating the cell-cell communications in clinical and *in vivo* models. Advances in humanized mouse models have closed this gap; however, the heterogeneity of the human xenografts grafted into the mouse models makes it challenging to study cell-cell communication in tumors. Primary human breast cancer cells have contributed to the advancement of our understanding of cancer biology, however, this system cannot evaluate interactions between carcinoma and intratumoral stromal cells. Co-culture compositions of two different cell type of cancer tissues *i.e*., breast cancer cells and stromal cells, were established in order to evaluate cell–cell interactions in tumor microenvironment. However, the use of random co-culture does not recreate the physiologically relevant interactions. Indirect co-culture method using transwell inserts provide valuable information on the role of secreted factors but the physical contact between the cells present in physiological conditions is missing. We and others have developed *in vitro* co-culture systems capable of engineering controlled environment of spatially defined micro-scale surfaces using microfabrication techniques^29^,^30^,^31^,^32^,^33^. This system provides an ideal platform to study the role of cell-cell communication on critical tissue function. In this study, we hypothesize that Src mediated trastuzumab resistance might be caused, at least in part, by cell-cell communication between MSCs and tumor cells, where in physical intimacy between the cells is the key factor. To test this hypothesis, we developed a co-culture system whereby HER-2 positive tumor cells are co-cultured with MSCs, and the ability of the MSCs to activate Src and modulate downstream drug resistance pathways is observed.

## Results

### Physical Contact of MSCs with Breast Cancer Cells Activates Src

**Figure 1:**
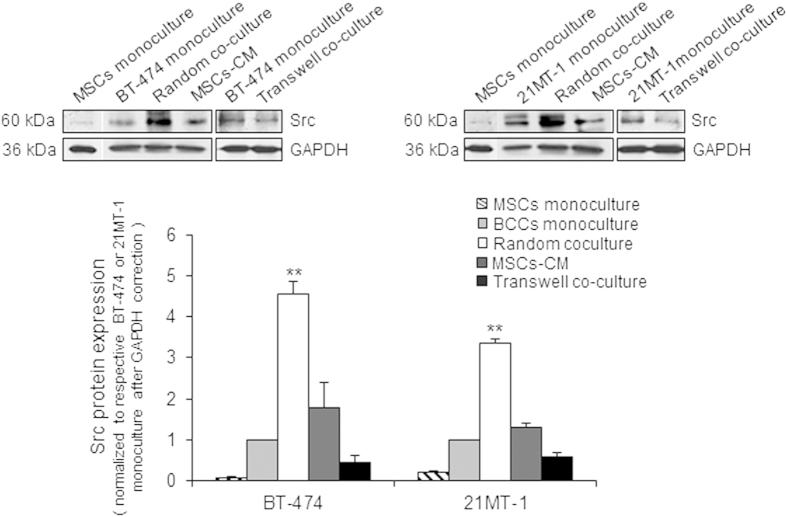
*Physical contact of MSCs with breast cancer cells activates Src*. (**a**) Upper panel shows representative immunoblots of Src detected in BT-474 and 21MT-1 cells co-cultured with MSCs, transwell co-culture or MSCs-CM. (**b**) Lower panel shows respective densitometry of bands; monocultures of BT-474 and 21MT-1 were used as control. Asterisks represent statistical significant differences in change in expression of Src in co-cultures samples compared with monoculture control. Data are mean ± SD; n = 3 independent experiments; **p < 0.01 compared with control.

To test the hypothesis that physical intimacy and not soluble factors from MSCs activates Src, we established four different systems to recreate these interactions. Human breast cancer cells were cultured 1) alone (monoculture); 2) treated with conditioned media from MSCs (MSCs-CM) to introduce secreted soluble factors into breast cancer cells; 3) co-culture of breast cancer cells with MSCs using transwell (Boyden Chamber) to monitor cell-cell interactions through soluble factors derived from each cells and 4) random co-culture with MSCs which contains the physical contact and ability to share soluble signaling factors (co-culture). We employed two developmentally distinct human breast cell lines for this study: 1) BT474 (HER2+ invasive breast cancer cells), and 2) 21MT-1 (stable patient-derived metastatic breast cancer cells isolated from the metastatic pleural effusion). We specifically chose 21MT-1 cells due to recent reports highlighting the 21T cell line’s ability to mimic *in vivo* cancer development and behavior within the *in vitro* enviroment through stage specific cell proliferation, migration, morpholgy, polarization, and gene expression profiles, and its consequent potential for usage as a valuable translational disease model for breast cancer^34^. A significant (p < 0.01) increase in Src protein expression was observed in random co-cultures of breast cancer cells and MSCs, while no significant change (p > 0.05) was observed in cells exposed to MSCs-CM, transwell co-culture, and monoculture of breast cancer cells (Fig. 1). Furthermore, no significant increase in Src expression was observed in breast cancer cells co-cultured with MCF10A cells (mammary epithelial cells), suggesting that this is a MSC-specific phenomena (Supplementary Fig. 1). This data suggest that the physical contact of breast cancer cells with MSCs is critical for MSCs mediated regulation of Src, and one of the possible mechanisms for Src activation in aggressive breast cancers.

### Establishing Patterned Co-culture of Breast Cancer Cells and MSCs

**Figure 2:**
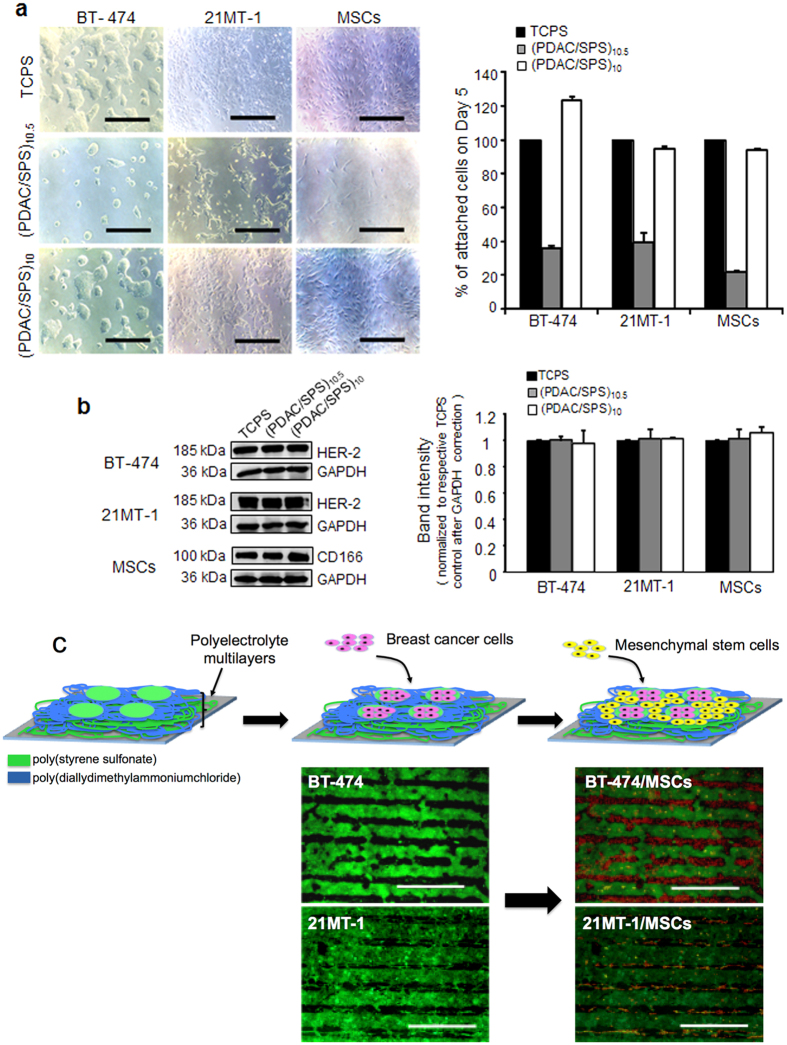
*Establishing patterned co-culture of breast cancer cells and MSCs*. (**a**) Left, optical micrographs of breast cancer cells and MSCs cultured on various surfaces after 5 days (Scale bar, 500 μm); Right, quantitative analysis of attachment of cells determined with MTT assay. Cells grown on TCPS were used as control. Mean ± SD; n = 3 independent experiments; **p < 0.01 compared with control. (**b**) Left, immunoblots show protein expression of specific markers of BT-474, 21MT-1 and MSCs on different surfaces. Right, respective densitometry of bands normalized with control after loading control (GAPDH) correction. Cells grown on TCPS served as control. (**c**) Scheme illustrating the approach for engineering patterned co-culture of breast cancer cells and MSCs (The scheme is not drawn to represent the true nature of the co-culture system) Fluorescent images of patterned monocultures of BT-474 and 21MT-1 (green) and patterned co-culture of breast cancer cells (green) and MSCs (red) after seeding MSCs (Scale bars- 200 μm).

To further investigate the effect of interaction of MSCs with breast cancer cells, we established a patterned co-culture system of breast cancer cells with MSCs using polyelectrolyte multilayer (PEM) films. We have previously screened several polymers similar to self-assemble protein network found in extracellular matrix^35^. Among all polymers, poly(diallyldimethylammonium chloride) (PDAC) and poly(4-styrenesulfonic acid) (SPS) were selected due to their ability to produce most consistent observations with cell attachment (Fig. 2A). For all experiments, 10 or 10.5 bilayers of PDAC/SPS were deposited on tissue culture polystyrene surfaces (TCPS) making SPS or PDAC as topmost layer and are designated as (PDAC/SPS)_10_ or (PDAC/SPS)_10.5_ respectively. No difference in morphology and growth of BT-474, 21MT-1 and MSCs were observed when cells were grown on (PDAC/SPS)_10_ compared to control. Quantitative analysis of attachment of cells on PEM films indicated higher attachment on (PDAC/SPS)_10_ compared to (PDAC/SPS)_10.5_ (Fig. 2A and Supplementary Fig. 2). The specific protein markers for breast cancer cells (HER-2 expression) and for MSCs (CD166) remained unchanged on both polymer surfaces compared to control, suggesting that polymer surfaces do not influence the biology of the cells beyond the cell adhesion behavior (Fig. 2B).

The approach for developing the patterned co-culture of breast cancer cells and MSCs consisted of capitalizing upon the cell adhesive and resistive property of SPS and PDAC, respectively, as shown in Fig. 2C and Supplementary 3. Unlike random co-culture where interaction of two cells is difficult to control, patterned co-culture provides a highly controlled system where cell placement and precise cell-cell interaction can be tuned by varying size and shape of patterns on fabricated surfaces. Patterns were fully characterized before seeding cells on fabricated surfaces using negatively charged fluorescent dye and polystyrene carboxylate microparticles (data not shown). Breast cancer cells preferentially attached on the SPS patterns when they were seeded on the PEM surfaces (Fig. 2C). MSCs were subsequently seeded onto the patterns of breast cancer cells thus engineering patterned co-cultures of breast cancer cells and MSCs (Fig. 2C). We also measured the average percent coverage of breast cancer cells and MSCs in 15 different patterns that demonstrated consistent patterning technique and development of patterned co-culture of breast cancer cells with MSCs (Supplementary Fig. 4).

### MSCs induce Src Activation in Patterned Co-culture and not in Conditioned Media

**Figure 3:**
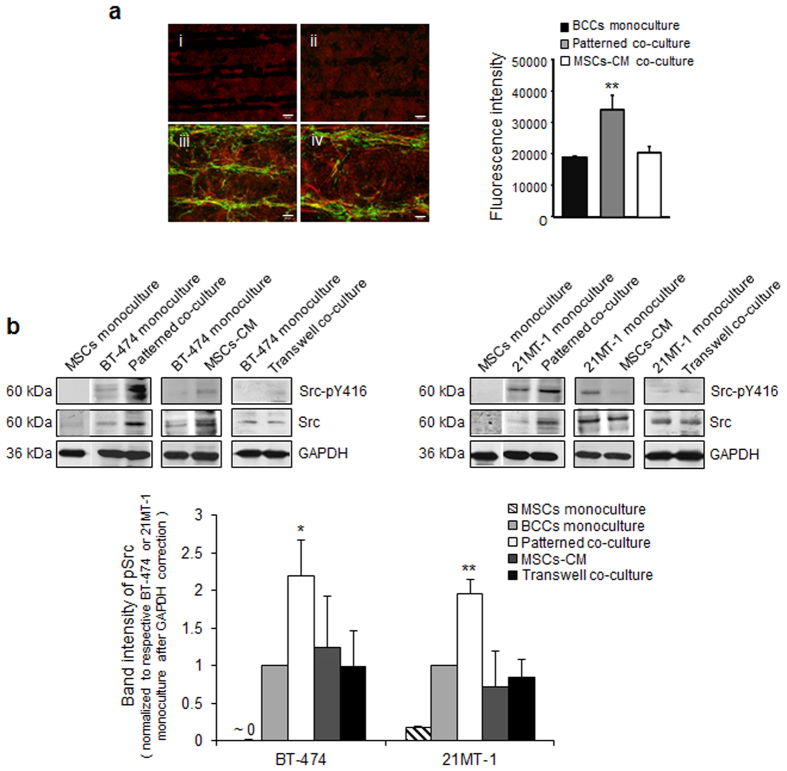
*MSCs induces Src activation in patterned co-culture and not in conditioned media*. (**a**) Immunostaining of Src (FITC, green) and MSCs marker- CD166 (Cy3, red) in (i) patterned monoculture of breast cancer cells; (ii) Breast cancer cells exposed to MSCs-CM; (iii) patterned co-culture of breast cancer cells with MSCs; (iv) enlarged image of (iii). (**b**) Upper panel, immunobots show activation of Src in breast cancer cells sorted after co-culture with MSCs using FACS, and in breast cancer cells exposed to MSCs-CM and in transwell co-culture. Lower panel, quantification of band intensity to assess the relative phosphorylation of Src at tyrosine 416 position (Y416). Mean ± SD; n = 3 independent experiments; *p < 0.05 compared with monoculture; **p < 0.01 compared with monoculture.

Given our observation that MSCs induce Src activation in random co-culture, we tested that phenomenon in our patterned co-culture system as well. To investigate this, we probed breast cancer cells and MSCs using antibodies against Src (red) and CD166 (green) (Fig. 3A). Akin to random co-culture, breast cancer cells in co-culture with MSCs indicated higher Src expression compared to monoculture and breast cancer cells exposed to conditioned media from MSCs (MSCs-CM) (Fig. 3A, upper panel). Quantification of fluorescence intensity shows that the Src expression in co-cultures increased ∼2 fold compared to the monoculture (Fig. 3A, lower panel). In co-culture, MSCs also show Src expression, however, merge images indicated that majority of the Src fluorescence is from the breast cancer cells.

To further investigate how physical contact of MSCs regulates Src activation in breast cancer cells, we examined whether MSCs in co-culture with breast cancer cells may activate Src tyrosine kinase. To quantify the Src expression in breast cancer cells, we sorted the co-culture cells using fluorescence activated cell sorting (FACS) by pre-staining the breast cancer cells and MSCs with red and green dye prior to co-culture. Src expression in FACS sorted cells was examined using western blotting. To validate the efficiency of FACS, the sorted cells were probed with breast cancer specific marker HER-2, and MSCs specific marker, CD166. Results indicated that the HER-2 expression and CD166 expression were observed only in sorted breast cancer cells and sorted MSCs, respectively (Supplementary Fig. 5). Compared to monoculture, transwell co-culture, and MSCs conditioned media treated breast cancer cells, patterned co-culture significantly (p < 0.05 and p < 0.01) increased Src phosphorylation at Y416 (Src-pY416) (Fig. 3B and Supplementary Fig. 5), an indicator of Src activity^6^,^7^,^36^. Together, our data indicate that physical contact of MSCs with breast cancer cells activates Src kinase activity in HER-2 overexpressing breast cancer cells, and this is not observed in breast cancer cells in mono-culture, transwell co-culture, or those treated with conditioned media from MSCs.

### MSCs Activates Pathways Regulating Tumor Growth and Trastuzumab Resistance

**Figure 4:**
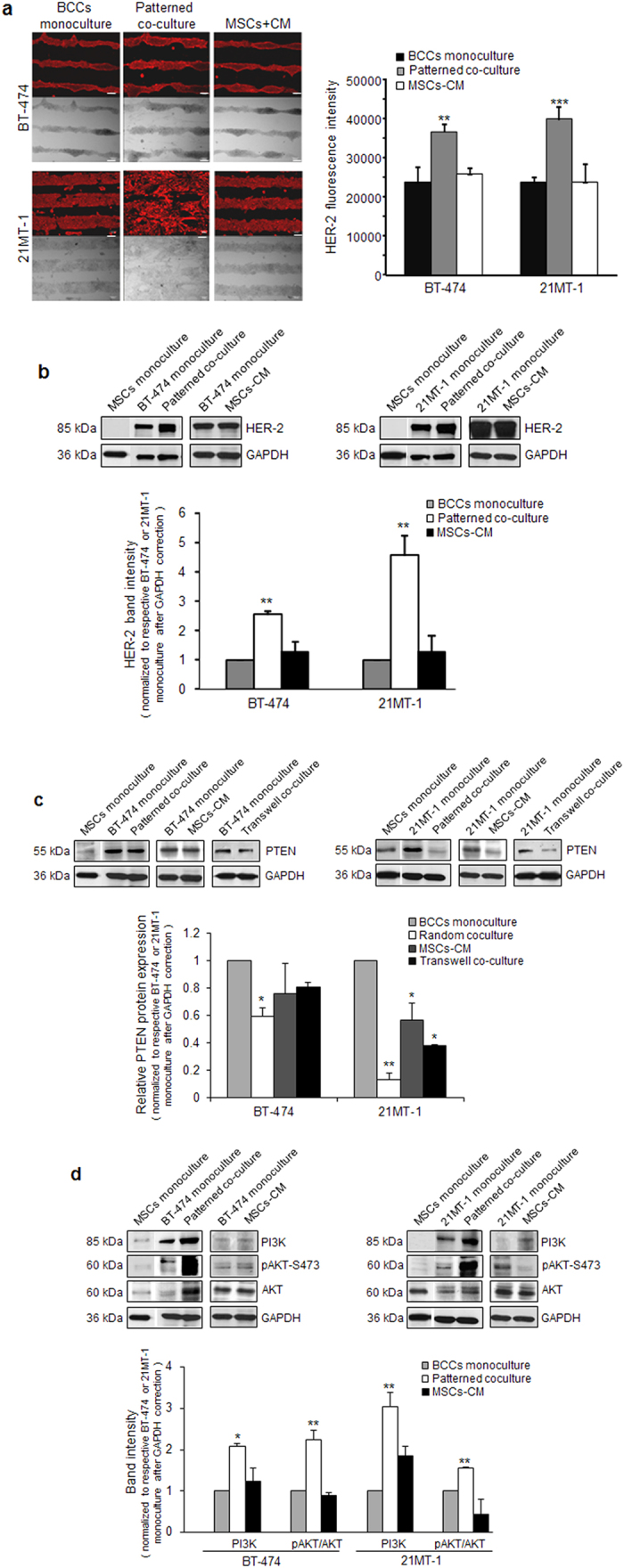
*MSCs activates pathways regulating tumor growth and trastuzumab resistance*. (**a**) Left, Immunostaining of HER-2 (Texas red) to assess the expression of HER-2 in patterned co-culture. Right, quantification of fluorescence intensity of HER-2 expression using imageJ. Mean ± SD; n = 3 independent experiments; **p < 0.01 compared with monoculture; ***p < 0.001 compared with monoculture. (**b**) Top, immunoblots of HER-2 expression in BT-474 and 21MT-1 cells sorted after co-culture with MSCs, and after exposed to MSCs-CM. Bottom, respective densitometry of HER-2 expression normalized with monoculture after GAPDH correction. Mean ± SD; n = 3 independent experiments; **p < 0.01 compared with monoculture. (**c**) Top, immunoblots of PTEN expression in BT-474 and 21MT-1 cells sorted after co-culture with MSCs, transwell co-culture and after exposed to MSCs-CM. Bottom, respective densitometry of PTEN expression normalized with monoculture after GAPDH correction. Mean ± SD; n = 3 independent experiments; **p < 0.01 compared with monoculture. (**d**) Top, western blot analysis of PI3K, pAKT and AKT. Bottom, respective densitometry of bands normalized with monoculture after loading control (GAPDH) correction. Expression of pAKT was further normalized with AKT (pAKT/AKT). Data are mean ± SD; n = 3 independent experiments; *p < 0.05 compared with monoculture; **p < 0.01 compared with monoculture.

Src activation has been demonstrated to play a direct role in the progression of aggressive HER-2 positive breast cancer^6^,^7^. To explore the effect of MSCs on tumor growth, we examined whether MSCs in contact with tumor cells has any effect on HER-2 expression, an important biomarker and indicator of tumor growth and progression^1^,^2^. Immunostaining of HER-2 demonstrated increased fluorescence in patterned co-culture as compared to the monoculture and cancer cells exposed to MSC conditioned media (Fig. 4A and Supplementary Fig. 4). Quantification of fluorescence intensity indicated higher HER-2 expression in breast cancer cells in co-culture compared to both monoculture and breast cancer cells exposed to MSCs-CM. To further confirm that MSCs mediated Src activation regulate HER-2, we examined the protein expression in breast cancer cells after co-culture using western blotting (Fig. 4B). A notably increased HER-2 expression in both BT-474 and 21MT-1 cells were observed in co-culture with MSCs compared to monoculture and MSCs-CM treated cells. Also, upregulation of HER-2 expression was more prominent in 21MT-1 cells, which are derived from metastatic cancer patients, suggesting the potential role of MSCs in more aggressive tumors.

To investigate how MSCs mediated activation of Src regulates phosphatase and tension homolog (PTEN), we examined whether breast cancer cells in co-culture with MSCs may inhibit PTEN, since Src activation has recently been reported to promote loss of PTEN^8^. We observed reduced expression of PTEN in BT-474 and complete loss of PTEN in 21MT-1 cells in co-culture with MSCs. In contrast, no change was observed in BT-474 cells exposed to MSCs-CM or in transwell co-culture, however, substantial downregulation was observed in 21MT-1 cells exposed to MSCs-CM and in transwell co-culture (Fig. 4C and Supplementary Fig. 5). This suggests that PTEN loss is not limited to the physical contact of MSCs with breast cancer cells in more aggressive cancers. This data is significant; as for the ***first time*** a PTEN deficient breast cancer *in vitro* model has been generated without the use of chemical inhibitors^37^,^38^, matrix stiffness^39^, or silencing RNAs^40^,^41^, thus indicating that our co-culture model is a physiologically relevant model. PTEN is a well-known tumor suppressor, and loss of PTEN has long been associated with tumor progression and drug resistance^42^. Furthermore, several reports in different tumor models have shown that PI3K/Akt signaling pathways become fundamental proliferative and survival pathways under PTEN loss or Src activation^8^,^43^. Thus, we investigated if physical contact of breast cancer cells with MSCs regulates these pathways. In our experimental settings, we observed ~2 fold increase in PI3K expression in BT-474 cells and ~3 fold increase in 21MT-1 cells when co-cultured with MSCs (Fig. 4D). Correspondingly, we observed activation of AKT by phosphorylation at serine 473 (AKT-pS473) in BT-474 as well as in 21MT-1. Significant increase (p < 0.01) in total AKT was also observed in BT-474 exposed to MSCs, in contrast no change in total AKT was observed in 21MT-1. Notably, no significant changes were observed in BT-474 and 21MT-1 cells exposed to MSCs-CM. These data are indicative of the role of MSCs mediated regulation of the proliferative and survival pathways of breast cancer cells where Src is activated and/or HER-2 is upregulated.

### Breast Cancer Cells in Co-culture with MSCs Contribute to Trastuzumab Resistance

**Figure 5:**
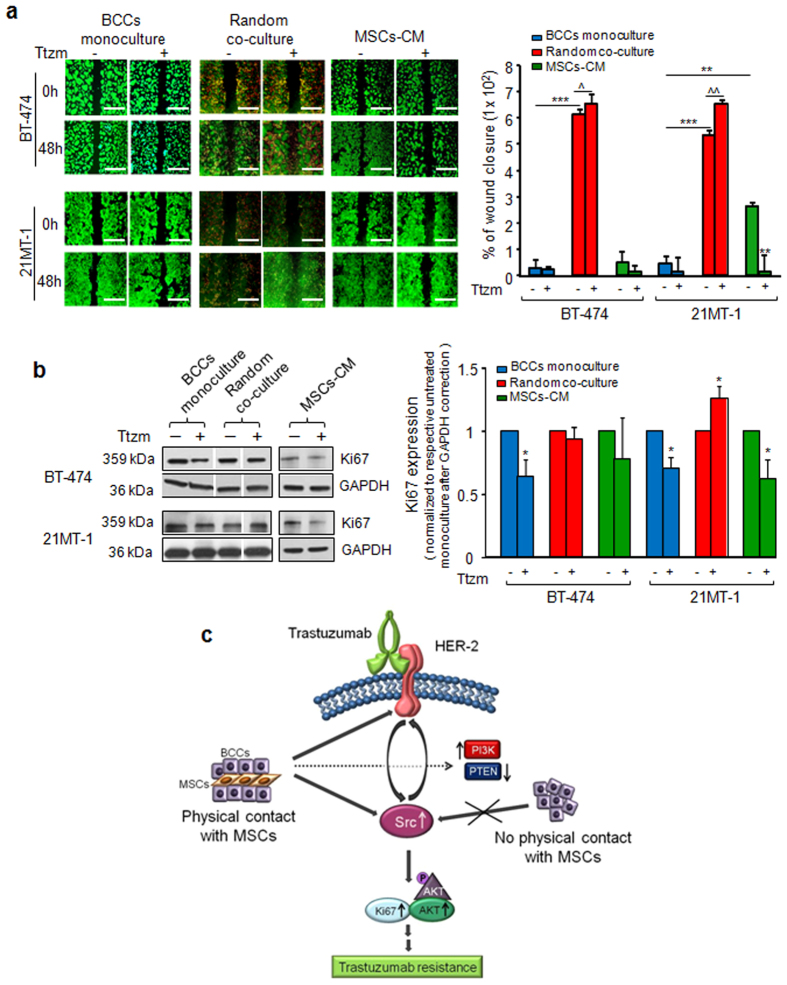
*Breast cancer cells in co-culture with MSCs contribute to trastuzumab resistance*. (**a**) Migration assay data detailing the migration of cells out from a confluent monolayer onto a featureless scratch or wound. Left, representative fluorescent images of breast cancer cells (CFDSE, green) and breast cancer cells co-cultured with MSCs (PKH26, red) before and after treatment with trastuzumab (Ttzm) at time = 0 h and 48 h. Scale bar 500 μm. Right, quantification of wound closure. The percentage of wound closure was determined by using following equation: % wound closure  =  [(Area @ t = 0–Area @ t = 48)/Area @ t = 0h]× 100%. Data are mean ± SD; n = 3 independent experiments; **p < 0.01 compared with untreated monoculture; ***p < 0.001 compared untreated monoculture; ^^p < 0.05 compared with random co-culture; ^p < 0.01 compared with random co-culture. (**b**) MSCs in co-culture with breast cancer cells confer trastuzumab resistance. Left, western blot analysis of Ki67 in breast cancer cells sorted by FACS after co-culture with MSCs, and breast cancer cells exposed to MSCs-CM in the presence or absence of Ttzm. Right, respective densitometry of bands normalized with respective untreated group after loading control (GAPDH) correction. Mean ± SD; n = 3 independent experiments; *p < 0.05 compared with untreated monoculture. (**c**) Suggested model of trastuzumab resistance mediated by MSCs via activation of Src. Figure drawn by Dr. Amita Daverey.

To investigate if the physical intimacy of MSCs may confer resistance to trastuzumab in breast cancer cells, we assessed the migratory potential of cancer cells in co-culture when exposed to the drug. Trastuzumab has been shown to inhibit cell migration in both *in vitro* and *in vivo* studies, however the drug loses this ability in drug resistant tumors^3^. In order to evaluate the effect of direct contact of MSC on breast cancer cells migration, we probed the migration capacity of BT-474 and 21MT-1 in mono-cultures, exposed with MSCs-CM, and co-cultures with MSCs in the presence of trastuzumab (Fig. 5A). A scratch was made in a sub-confluent cell monolayer and cells were allowed to migrate into the cell-free area. The distance moved by the cells in control, MSC-CM treated cells, and co-cultured plates, respectively, was compared when exposed to the drug. To distinguish the cell populations, breast cancer cells and MSCs were stained with green dye, CFDSE and red dye, PKH26 respectively. In the absence of trastuzumab, the co-culture of BT-474 and 21MT-1 cells with MSCs showed significantly (p < 0.001) higher migration after 48 h of co-culture as compared to the respective monocultures. On the other hand, only 21MT-1 cells treated with MSCs-CM demonstrated significant migration as compared to monoculture (p < 0.05). When BT-474 mono-culture cells were exposed to Ttzm, there was no significant change in the migratory property in mono-culture and MSCs-CM conditions but BT-474 in co-culture showed significantly (p < 0.01) higher migration when compared to untreated co-culture samples. In contrast, when 21MT-1 mono-culture and MSC-CMs cells were exposed to Ttzm, there was no significant no signficiant inhibition in the migratory potential while 21MT-1 in co-culture showed significantly (p < 0.05) even after exposed to Ttzm. This data suggest that direct interaction of MSCs with breast cancer cells promotes migration of breast cancer cells and potentially regulates the Ttzm responsiveness in breast tumor cells when in physical contact.

In the light of this data, we further validated the effect of MSCs-breast cancer cells interaction on trastuzumab resistance by determining Ki67, a clinical maker for the rate of proliferation in breast cancer cells and key hallmark of trastuzumab mechanism of action (Fig. 5B and Supplementary Fig. 6). Breast cancer cells were cultured as monocultures, co-cultures with MSCs, and MSC-CM treated groups in the presence or absence of trastuzumab for 48 h. Breast cancer cells in the co-culture system were separated using FACS and probed for Ki67, a proliferation marker. The Ki67 expression decreased significantly (p < 0.05) in BT-474 and 21MT-1 monocultures after trastuzumab treatment compared to untreated monocultures. However, no change in Ki67 expression was observed in BT-474 and 21MT-1 co-cultured with MSCs after trastuzumab treatment, thus indicating that the tumor cells are unresponsive to the drug. Interestingly, no significant effect was observed in Ki67 expression when BT-474 exposed to MSCs-CM treated with Ttzm but 21MT-1 exposed to MSCs-CM treated with Ttzm indicated decreased Ki67 expression. The differential behavior between Bt-474 and 21MT-1 might potentially be due to the different sources of the tumor cells, which needs further investigation.

## Discussion

Trastuzumab is a highly specific, targeted cancer therapy that has brought valuable therapeutic benefits to HER-2-overexpressing cancer patients^44^. However, the limited response and drug resistance, even in patients with tumors expressing very high levels of HER-2, raises questions on the mechanisms of trastuzumab resistance in patients. The possible mechanisms that contribute to the frequent development of resistance to trastuzumab are at its infant stages, and are an active area of investigation^4^,^8^. Recent studies have demonstrated that activation of Src is important during development of trastuzumab resistance. Here, we report that the breast cancer cell in physical contact with MSCs elicit Src activation and regulates downstream trastuzumab resistance pathways (Fig. 5C). We developed a co-culture model to study the interaction between MSCs and breast cancer cells, and identified previously unexplored regulation of Src through MSCs in HER-2 positive breast cancer cells. The unique patterned co-culture model enables us to recreate the cell-cell communication underway in tumor development and progression. The model provides the ability to capture the temporal changes in the cell biology as the tumor develops through various stages. The significant advantages of our patterned co-cultures are: (1) the protein-free cell-culture environment reduces nonspecific function; (2) provides ease of visualization, patterned co-culture platform has several advantages over random co-cultures; (3) precise and consistent control of degree of contact between the cells (demonstrated in Supplementary Figure 4); and (4) the cancer cell-MSC interaction can be controlled by varying the percent coverage of the cells using different pattern dimensions to emulate different stages of a breast tumor. To validate the physiological relevance of this study, we used patient-derived HER-2 positive metastatic breast cancer cells (21MT-1) isolated from the metastatic pleural effusion. This finding led to the new concept that physical contact of MSCs is a key factor that contributes to trastuzumab’s resistance by activating Src. We also found that MSCs presence causes overexpression of HER-2 and loss of PTEN. This indicates that MSCs regulates the functional crosstalk between the HER-2 receptor and the PTEN tumor suppressor in breast cancer cells via Src. Src plays important oncogenic functions (e.g., inactivates PTEN) when bound to and activated by overexpressed HER-2, which contributes to the trastuzumab resistance. To our knowledge, no study has specifically investigated the role of MSCs in regulation of Src in breast cancer, which has been shown as common node for multiple pathways associated with growth, invasion and trastuzumab resistance of this disease.

*In vitro* and *in vivo* studies have demonstrated the role of Src in cellular growth and proliferation, angiogenesis, invasion and metastasis. The elevated expression of Src has been previously reported in majority of breast cancer tissues which is significantly associated with HER-2 status and metastatic disease^6^,^7^,^45^. Belsches-Jablonski *et al*. identified stable complex formation between Src and HER-2 in 13 breast cancer cell lines^46^. On the other hand role of MSCs on breast cancer progression remain unclear and controversial. It has been reported that bone marrow derived human MSCs can stimulate MCF-7 breast cancer cell proliferation *in vitro* and promote breast tumor metastasis in a xenograft model^15^,^16^,^17^. Recently, Clarke *et al*. showed the inhibition of migration and invasion in breast cancer cells, MDA-MB-231 and T47D, through immortalized MSC line RCB2157^47^. Of note, they used co-cultured breast cancer cells in a transwell insert above MSCs or MSCs conditioned media, therefore, results obtained here are mediated only through soluble factors. However, there are several reports that show that MSCs inhibited the growth of tumors in xenograft breast cancer model when co-inoculated with human cancer cells or administered intravenously to animals^48^,^49^. In contrast, Usha *et al*. documented that MSCs do not promote or inhibit the tumor growth and development in a clinically relevant mouse model^50^. We observed MSCs resulted in the overexpression of HER-2 expression in breast cancer cells in our co-culture model, however conditioned media or transwell co-culture did not have any effect on the HER-2 expression. This observation is corresponding to the Src activation, thus, increased expression of HER-2 and Src may have direct interaction which results in promoting the growth of breast cancer cells. Our study showed increased proliferation of breast cancer cells in co-culture systems as evident by increased expression of Ki67. Therefore, from our study and previous reports it is evident that the outcome of MSCs on tumor growth and progression is dependent on the tumor types, the sources of MSCs and mouse models such as syngeneic versus xenogeneic graft.

Several studies based on pharmacological inhibitors of Src family tyrosine kinases indicate that Src activation is upstream of PI3K. In addition, PI3K has been shown to be a preferential substrate for Src and a major signaling hub downstream of HER-2. We demonstrated that activation of Src at Tyr416 not only activates AKT (Ser473) but also increased the activity of PI3K in breast cancer cells co-cultured with MSCs. In support to our findings, recent report has reported the activation of PI3K/AKT signaling pathways mediated by Src activation in trastuzumab resistant breast cancer cells^8^. In addition, their data showed that increased Src phosphorylation at Tyr416 regulates the loss of PTEN. Interestingly, we observed PTEN loss in highly metastatic cell line 21MT-1 mediated through physical contact of MSCs with breast cancer cells as well as through soluble factors alone. In contrast, PTEN loss observed in BT-474 cells was primarily due to result of physical contact of MSCs with breast cancer cells. In contrast, Dasari *et al*. observed that the MSCs are able to upregulate PTEN simultaneously downregulating the PI3K/Akt pathway^51^. However, these studies were carried out on glioblastoma and human umbilical cord blood-derived MSCs which have possibly have a different synergistic pathway compared to breast tumors. To our knowledge, this is the ***first report*** that showed PTEN loss by MSCs in breast cancer cells without the use of chemical inhibitors^37^,^38^, matrix stiffness^39^, or silencing RNAs^40^,^41^. Since Src Tyr416 served as a direct substrate for PTEN loss, it is possible that MSCs activates Src via knocking down the PTEN. However, we did not observe activation of Src in 21MT-1 through conditioned media or transwell co-culture, thus, whether MSCs regulate Src independently or through PTEN loss remains to be explored. Earlier studies showed that loss of PTEN, Src activation and increased activity of PI3K/AKT pathways confer trastuzumab resistance to breast cancer cells^8^,^42^. In support of these studies, our wound healing data indicate that breast cancer cells harbor resistant to trastuzumab therapy in the presence of MSCs which was further confirmed by increased proliferation of breast cancer cells in co-cultures as compared to mono-culture systems.

In summary, our study identifies a systemic mechanism of resistance via interaction of breast cancer cells with MSCs by activation of Src and its downstream PI3K/AKT pathways. We have provided evidence that the physical contact is important for breast cancer cells to develop drug resistance and enhance tumor growth, metastasis and drug resistance. To validate the physiological relevance of this study, we used patient-derived HER2+ metastatic breast cancer cells (21MT1) isolated from the metastatic pleural effusion. Our findings introduce important players to the field of drug resistance, and indicate targeting physical interaction of MSCs and breast cancer cells might be a possible strategy for drug resistant HER-2 positive breast tumors. It should be noted that the MSC-mediated Src activation and trastuzumab resistance mechanism detailed here is potentially one of the many possible tumor microenvironment-mediated drug resistance interactions. Our findings point to the tumor microenvironment as an important but understudied source of anticancer drug resistance. Moreover, the results suggest that such resistance mechanisms can be uncovered through the systematic dissection of interactions between tumors and their microenvironment. Future studies should therefore seek to identify such resistance mechanisms for all of the drugs that are in development, potentially leading to mechanism-based combination regimens to overcome drug resistance.

## Materials and Methods

HER-2 overexpressed breast cancer cell lines, BT-474 and 21MT-1 were kindly provided by Drs. Hamid Band and Vimla Band (UNMC, USA). Adipose derived MSCs were obtained from American Type Culture Collection (ATCC). BT-474 cells were grown in complete α-MEM made up of α-MEM medium supplemented with 5% FBS, 1% L-glutamine, 100 U/mL penicillin G, 100 μg/mL streptomycin, 20 mM HEPES, non-essential amino acids and sodium-pyruvate (complete α-MEM). 21MT-1 cells were developed by Dr. Vimla Band from a metastatic breast cancer patient and maintained in complete α-MEM, further supplemented with 12.5 ng/ml epidermal growth factor (EGF) and 1 ng/ml hydrocortisone. MSCs were grown in MSCGM (Lonza) supplemented with growth factors provided with kit. For all experiments MSCs were not used more than 3 passages. All cells were grown in the presence of 5% CO2 at 37 °C.

Two co-culture systems were utilized: (1) direct co-culture (random or patterned co-culture), (2) indirect co-culture (conditioned media from MSCs or transwell co-culture). For random co-culture, 0.3 × 10^6^ BCCs were seeded into per well of 6 well plate followed by seeding 0.1 × 10^6^ MSCs on Day 2. BCCs were allowed to grow for 72 h in the presence of MSCs. For patterned co-culture, 0.6 × 10^6^ BCCs were first seeded into per well of fabricated 6-well plate and allowed to grow for two days. After that, 0.2 × 10^6^ MSCs were added on day 2, cells were were allowed to grow for 72 h in co-culture. For conditioned media (MSCs-CM) experiments, 0.5 × 10^6^ MSCs were seeded into 60 mm petri dish and allowed to grow for 2 days. Conditioned media was collected from 80% confluent monolayer at passage 3 on Day 2, and store at −80 °C until use. MSCs-CM was added to the BCCs cultures on day 2 and allowed to expose to the MSCs-CM for 72 h. For transwell experiments, 30,000 BCCs per well were first seeded on the bottom of 24-well transwell cell culture system (Pore size 0.4 μm; Costar Corp, USA) and allowed to grow for two days. On day 2, transwell insert were placed into wells of BCCs cultures, and 10,000 MSCs per well were seeded onto the membrane of transwell cell culture inserts. The cultures were allowed to grow for 72 h. For all co-culture systems, medium of BCCs was exchanged with co-culture media (1:1 ratio of BCCs medium and MSCs medium) on day 2. For all experiments monoculture of BCCs grown in co-culture media was used as control.

### Wound healing assay

Cells were seeded as monocultures and co-cultures in a 6 well plate as described earlier in “co-culture” section. Cells were allowed to grow under permissive conditions until 80% confluence and a wound was created using a sterile 10 μl pipette tip. The wounded cells were washed three times with PBS to remove detached cells and the cells were incubated in co-culture media in the presence or absence of 20 μg/ml trastuzumab, a kind gift from Genentech (South San Francisco, CA). The wound area at 0, 24 and 48 h after scratching was photographed using a microscopy system (Axiovert40 Zeiss, Germany), and the cell-free wound area was measured using NIH Image J software. The percentage of wound closure was determined by using following equation:

% of wound closure = [(Area @ t = 0–Area @ t = 48)/Area @ t = 0h] × 100%

### Statistics

Statistical analysis were made using GraphPad Instat 5.0 program (GraphPad Software, USA). Comaprison between each groups were determined using nonparametric One-Way-ANOVA and Student-Newman-Keuls Multiple Comparisons Test. Each value represents mean ± sd from minimum 3 biological replicates. Statistical significance between two samples was determined by a P value of less than 0.05. P values of less than 0.05, 0.01, or 0.001 are described as *P < 0.05, **P < 0.01, or ***P < 0.001, respectively.

Please refer to SI Materials and Methods for detailed methods.

## Additional Information

**How to cite this article**: Daverey, A. *et al*. Physical Intimacy of Breast Cancer Cells with Mesenchymal Stem Cells Elicits Trastuzumab Resistance through Src Activation. *Sci. Rep*. **5**, 13744; doi: 10.1038/srep13744 (2015).

## Supplementary Material

Supplementary Information
