# Supplementary material for: Physical Intimacy of Breast Cancer Cells with Mesenchymal Stem Cells Elicits Trastuzumab Resistance through Src Activation
Source: Sci Rep. 2015 Sep 8;5:13744. doi: 10.1038/srep13744 (PMC4561910; doi:10.1038/srep13744)
Supplement: Supplementary Information [file srep13744-s1.pdf]

Physical Intimacy of Breast Cancer Cells with Mesenchymal Stem Cells Elicits Trastuzumab Resistance through Src Activation

Amita Daverey[1], Allison P Drain[1], Srivatsan Kidambi[1,2,3#]

[1]Department of Chemical and Biomolecular Engineering,

University of Nebraska-Lincoln, NE, 68588

[2]Nebraska Center for Materials and Nanoscience,

University of Nebraska-Lincoln, NE, 68588

[3]Regenerative Medicine Program,

University of Nebraska Medical Center, NE, 68198.

[#]indicates corresponding author. Email: skidambi2@unl.edu

SUPPLEMENTARY INFORMATION

Materials

Poly(dimethylsiloxane) (PDMS) from the Sylgard 184 silicone elastomer kit (Dow Corning, Midland, MI) was used to prepare stamps. Poly(diallyldimethylammoniumchloride) (PDAC) (Mw~100,000-200,000) as a 20 wt % solution, sulfonated poly(styrene), sodium salt (SPS) (Mw~70,000), sodium chloride, bovine serum albumin (BSA), PKH26 cell staining kit, carboxyfluorescein succinimidyl ester (CFSE), carboxyfluorescein diacetate succinimidyl ester fluorescent dyes (CFDSE) and alpha-Minimum Essential Medium (a-MEM) were purchased from Sigma-Aldrich Corporation (St. Louis, MO, USA). Fetal bovine serum (FBS), penicillin-streptomycin, L-glutamin, 4-(2-hydroxyethyl)-1-piperazineethanesulfonic acid (HEPES), Non-essential amino acids and sodium-pyruvate were purchased from Life Technologies (Gaithersburg, MD). HER-2 polyclonal antibody was purchased from Abcam (Cambridge, MA). All other antibodies used in this study were obtained from Millipore/Chemicon (Billerica, MA, USA). Horseradish peroxide (HRP)-conjugated IgGs were purchased from Santa Cruz Biotechnology, Inc. (Santa Cruz, CA, USA).

Cell lines and culture conditions

HER-2 overexpressed breast cancer cell lines, BT-474 and 21MT-1 were kindly provided by Dr. Hamid Band (UNMC, USA). Adipose derived MSCs and MCF10A cells were obtained from American Type Culture Collection (ATCC). BT-474 cells were grown in complete α-MEM made up of α-MEM medium supplemented with 5% FBS, 1% L-glutamine, 100 U/mL penicillin G, 100 μg/mL streptomycin, 20mM HEPES, non-essential amino acids and sodium-pyruvate (complete α-MEM). 21MT-1 cells were developed by Dr. Vimla Band from a metastatic breast cancer patient and maintained in complete α-MEM, further supplemented with 12.5 ng/ml epidermal growth factor (EGF) and 1 ng/ml hydrocortisone. MSCs were grown in MSCGM (Lonza) supplemented with growth factors provided with kit. For all experiments MSCs were not used more than 3 passages. MCF-10A cells were maintained in phenol red free DMEM/F12 culture medium supplemented with sodium bicarbonate (1200 mg/L), 5% horse serum, insulin (10 μg/ml), L-glutamine (2 mM), penicillin G (100 U/mL), streptomycin (100 mg/mL), EGF (20 ng/ml), hydrocortisone (500 ng/ml), and cholera toxin (100 ng/ ml). All cells were grown in the presence of 5% CO2 at 37 ^{o}C.

Cell adhesion on polymer surfaces

Polyelectrolyte Multilayers (PEMs) were built on tissue culture polystyrene surfaces (TCPS) using PDAC and SPS polymers as describes earlier.[1-3] Briefly, 0.1M concentration Both polymer dipping solutions were prepared in deionized (DI) water with 0.02M and 0.01M concentration of PDAC and SPS solutions were used for engineering the thin films. TCPS were treated using a Harrick plasma cleaner (Harrick Scientific Corporation, Broading Ossining, NY) for five minutes. The plate was immediately inserted into a Carl Zeiss slide stainer to carry out the layer-by-layer assembly process. The plate was alternately dipped in PDAC and SPS solutions for 20 minutes with two 5 minute washing steps in DI water with agitation in between. Experiments were performed using ten and ten-and-a-half bilayers, $(PDAC/SPS)_{10}$ and $(PDAC/SPS)_{10.5}$, labeled as SPS and PDAC respectively. Prior to cell culture, fabricated surfaces were sterilized for overnight under UV. Breast cancer cells and MSCs were grown on SPS, PDAC and uncoated TCPS (control) surfaces for 5 days. Morphology of cells was assessed by optical microscopic images of the cells on different surfaces acquired with Axiovert40 Zeiss (Germany) inverted microscope for five days. MTT assay was performed on days 1, 3, and 5 as described earlier to quantify the attachment of cells on different surfaces.[4] Results presented represent triplicate experiments and are expressed as Mean ± SEM.

Fabrication of patterned surfaces

Line patterns were created on PEMs built on TCPS surfaces using microcontact printing. For that PDMS stamps were first prepared using the Sylgard elastomer kit by mixing a 12:1 polymer to cross-linker ratio. The stamps were plasma treated and inked with PDAC or SPS solutions depending on the patterns to be generated. The stamp was left in place for twenty minutes before being carefully removed and the surfaces were rinsed with DI water to remove loosely bound molecules. The patterned fabricated surfaces were subjected to the UV light overnight to ensure sterility before cell culture.

For cell patterns, breast cancer cells (BBCs) and MSCs were distinguished from one another by staining with carboxyfluorescein diacetate succinimidyl ester (CFDSE) dye (green) and PHK26 dye (red), respectively. Cells were trypsinized and washed with medium without serum, and incubated in 10 mg/mL CFDSE in PBS solution at a concentration of 1 x 10^7 cells/mL or in 2 X 10^{-6} M PKH26 in diluent C at a concentration of 1 x 10^7 cells/mL for 10 min at room temperature. Both staining reactions were quenched with the addition of an equal volume of serum and washed three times with medium. After final wash, cells were suspended in medium, and 0.6 x 10^6 BCCs were seeded first. On day 2, 0.2 x 10^6 MSCs were seeded. Fluorescent images of the cells patterns were acquired with Axiovert40 Zeiss (Germany) inverted microscope before and after co-culture with MSCs. To determined cell coverage area of BCCs and MSCs in patterned co-culture, area covered by BCCs or MSCs in patterned co-culture was measured with ProgRes CaptureProv2.8.0 (Zeiss) software provided by Axiovert40 Zeiss inverted microscope. The coverage area of BCCs or MSCs to total cell area was determined and expressed as a percentage.

Co-culture Experiments

Two co-culture systems were utilized: (1) direct co-culture (random or patterned co-culture), (2) indirect co-culture (conditioned media from MSCs or transwell co-culture). For random co-culture experiments, 0.3x10^6 BCCs were seeded per well of 6 well plate followed by seeding 0.1x10^6 MSCs on Day 2. BCCs were allowed to grow for 72 h in the presence of MSCs or MCF10A. For patterned co-culture experiments, 0.6x10^6 BCCs were first seeded per well of 6-well plates containing our polymer surfaces and allowed to grow for two days. After that, 0.2 x10^6 MSCs were added on day 2, cells were allowed to grow for 72 h in co-culture. For conditioned media (MSCs-CM) experiments, 0.5 x10^6 MSCs were seeded into 60 mm petri dish and allowed to grow for 2 days. Conditioned media was collected from 80% confluent monolayer at passage 3 on Day 2, and store at -80°C until use. MSCs-CM was added to the BCCs cultures on day 2 and allowed to expose to the MSCs-CM for 72 h. For transwell experiments, 30,000 BCCs per well were first seeded on the bottom of 24-well transwell cell culture system (Pore size 0.4 μm; Costar Corp, USA) and allowed to grow for two days. On day 2, transwell insert were placed into wells of BCCs cultures, and 10,000 MSCs per well were seeded onto the membrane of transwell cell culture inserts. The cultures were allowed to grow for 72 h. For all co-culture systems, medium of BCCs was exchanged with co-culture media (1:1 ratio of BCCs medium and MSCs medium) on Day 2. For all experiments monoculture of BCCs grown in co-culture media was used as control.

Fluorescence-activated cell sorting (FACS)

In direct co-culture system, BCCs were separated from MSCs by FACS Aria II flow cytometer (BD Bioscience, San Jose, CA). For sorting of cells, BCCs and MSCs were stained with CFDSE and PKH26 dyes respectively before seeding as described earlier in cell staining section. Cells were trypsinized after 72 h of co-culture, washed once with cold PBS followed by suspending cell pellet in 0.5 ml of PBS containing 10% FBS. The population of BCCs and MSCs was gated in CDFSE positive and PKH26 positive cell quadrant respectively set by monocultures of BCCs

and MSCs. The cells were sorted in exclusion mode and collected into sorting tubes. Minimum 30,000 sorted events were performed for each sample. For control experiment, instead of co-culturing BCCs and MSCs, stained BCCs and MSCs were mixed just prior to running the samples on flow cytometer. After collection of BCCs and MSCs in different tubes, the cells were centrifuged and cell pellet was obtained for western-blot analysis.

Western-blot analysis

For random co-culture cell, cells were washed three times with ice cold-phosphate buffered saline (PBS) and scraped in RIPA buffer (100 mM Tris, 5 mM EDTA, 5% NP40, pH-8.0) containing protease inhibitors cocktail (PIC) and phenylmethylsulfonyl fluoride (PMSF) followed by 10 min incubation on ice with intermittent vortexing. For patterned and indirect (MSCs-CM) co-cultures, cells were trypsinized and cell pellet was washed three times with ice-cold PBS. After that, cell pellet was dissolved in RIPA buffer containing PIC and PMSF followed by 10 min incubation on ice with intermittent vortexing. Clear cell lysate from all co-culture systems was obtained by centrifugation at 10,000 rpm for 10 min at 4°C and stored at -80 ^{o}C until use. Protein concentration was determined using Coomassie Plus Assay reagent purchased from Pierce (Rockford, IL). 10 mg of total protein was separated by 7.5% SDS-polyacrylamide gel electrophoresis and transferred to Immobilon P membranes (Millipore, Ballerica, MA) using transfer buffer (25 mM Tris, 192 mM glycine, 10% methanol). Membranes were blocked with 5% skimmed milk for 3 h at room temperature (RT) thereafter membranes were incubated with primary antibodies for overnight at 4 ^{o}C. Anti-pSrc (1:1000), anti-Src (1:1000), anti-HER-2 (1:1000), anti-Ki67 (1:1000), anti- PI3K (1:1000), anti-PTEN (1:1000), anti-pAKT (1:1000), anti-AKT (1:1000), anti-GAPDH (1:4000) or anti-CD166 primary antibodies were used followed by incubation with corresponding horse peroxidase-conjugated secondary antibodies (1:5000) at room temperature (RT) for one hour. Signal was detected using ECL and exposure to ECL Hyperfilm (Pierce, Rockford, IL). Densitometry was performed using Image Studio™ Lite Software ver 4.0 (LI-COR Biosciences).

Immunostaining

For immunofluorescence staining, cultures were washed 3 times with PBS, fixed in 4% paraformaldehyde in PBS at RT for 20 min, washed three times with PBS, followed by addition of 0.2% Triton X-100 in PBS to permeabilize cells for intracellular staining. Cultures were incubated overnight at 4 ^{o}C with anti-Src (1:1000), anti-CD166 (1:1000) or anti-HER-2 (1:1000). After that, cultures were washed three times with PBS and incubated with corresponding secondary antibodies (anti-rabbit FITC, anti-mouse Cy3 or anti-rabbit rhodamine conjugated) at RT for 1 h. Before taking images, cultures were washed three times with PBS and analyzed using an inverted (Olympus IX 81) confocal Microscope. Fluorescence intensity was measured using NIH Image J software.

Wound healing assay

Cells were seeded as monocultures and co-cultures in a 6 well plate as described earlier in "co-culture" section. Cells were allowed to grow under permissive conditions until 80% confluence and a wound was created using a sterile 10 µl pipette tip. The wounded cells were washed three times with PBS to remove detached cells and the cells were incubated in co-culture media in the presence or absence of 20µg/ml trastuzumab, a kind gift from Genentech (South San Francisco, CA). The wound area at 0, 24 and 48h after scratching was photographed using a microscopy system (Axiovert40 Zeiss, Germany), and the cell-free wound area was measured using NIH Image J software. The percentage of wound closure was determined by using following equation:

% of wound closure = [(Area @ t=0 – Area @ t=48)/Area @ t=0h] x 100 %

Statistics

Statistical analysis were made using GraphPad Instat 5.0 program (GraphPad Software, USA). Comparison between each groups were determined using nonparametric One-Way-ANOVA and Student-Newman-Keuls Multiple Comparisons Test. Each value represents mean ± sd from minimum 3 biological replicates. Statistical significance between two samples was determined by a P value of less than 0.05. P values of less than 0.05, 0.01, or 0.001 are described as $*P < 0.05$, $**P < 0.01$, or $***P<0.001$, respectively.

Supplementary Figure Legends

Supplementary Figure 1: *Expression of Src in random co-culture of breast cancer cells (BCCs) and MCF10A*. Upper panel, representative immunoblots of Src detected in BT-474 and 21MT-1 cells co-cultured with MCF10A. Lower panel, densitometry of bands normalized with respective monocultures of BT-474 or 21MT-1. Data are mean ± SD; n=3 independent experiments.

Supplementary Figure 2: *Attachment of BCCs (BT-474 and 21MT-1) and MSCs on fabricated surfaces*. Attachment of cells on $(PDAC/SPS)_{10.5}$ and $(PDAC/SPS)_{10}$ surfaces was determined with MTT on day 1, day 3 and day 5. Cells grown on TCPS were used as control. Mean ± SD, n=3 independent experiments.

Supplementary Figure 3: *Phase and Fluorescent images of patterned co-culture of BCCs (green) and MSCs (red)*. Upper panel, phase images of BT-474 and MSCs co-culture (i), monochrome images of BT-474 (ii) and MSCs (iii), and merged image shows patterned co-culture of BT-474 and MSCs (iv). Lower panel, phase images of 21MT-1 and MSCs co-culture (v), monochrome images of 21MT-1 (vi) and MSCs (vii), and merged image shows patterned co-culture of 21MT-1 and MSCs (viii). Scale bar 500 μm (ii, iii, iiv, vi, vii, viii) and 200 μm (i, v).

Supplementary Figure 4: *Percentage area coverage of BCCs and MSCs in patterned co-culture.* Area covered by breast cancer cells and MSCs was determined with software ProgRes CaptureProv3.8.0 (Zeiss) provided by Axiovert40 Zeiss inverted microscope.

Supplementary Figure 5: *Representative Immunoblots of the indicated proteins from patterned co-culture of BCCs and MSCs after FACS.* (**a**) Immunoblots show activation of Src and downregulation PTEN in BT-474 cells sorted out from patterned co-culture of BT-474 and MSCs. (**b**) Immunoblots show activation of Src and downregulation PTEN in 21MT-1 cells sorted out from patterned co-culture of 21MT-1 and MSCs. Efficiency of FACS was determined by probing the blots with breast cancer cells specific marker, HER-2, and MSCs specific marker, CD166. GAPDH was used as loading control.

Supplementary Figure 6: *Representative Immunoblots of Ki67 from random co-culture of BCCs and MSCs, and breast cancer cells treated with MSCs-CM.* (**a**) Left, western blot analysis of Ki67 in BT-474 cells sorted by FACS after co-culture with MSCs in the presence or absence of trastuzumab (Ttzm). Right, BT-474 cells exposed to MSCs-CM in the presence or absence of Ttzm. (**b**) Left, western blot analysis of Ki67 in 21MT-1 cells sorted by FACS after co-culture with MSCs in the presence or absence of Ttzm. Right, 21MT-1 cells exposed to MSCs-CM in the presence or absence of Ttzm.

Supplementary Figure 7: Full blots of figure 1a.

Supplementary Figure 8: Full blots of figure 2b.

Supplementary Figure 9: Full blots of figure 3b.

Supplementary Figure 10: Full blots of figure 4b.

Supplementary Figure 11: Full blots of figure 4c.

Supplementary Figure 12: Full blots of figure 4d.

Supplementary Figure 13: Full blots of figure 5b and supplementary figure 6.

Supplementary Figure 14: Full blots of supplementary figure 1.

Supplementary Figure 15: Full blots of supplementary figure 5a.

Supplementary Figure 16: Full blots of supplementary figure 5b.

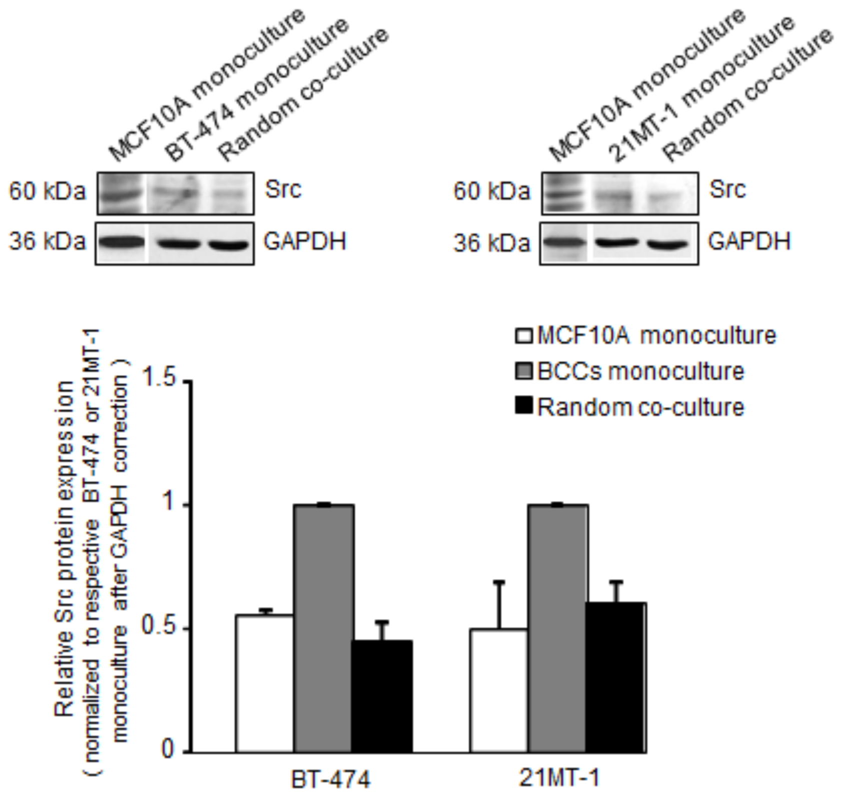

Supplementary Figure 1: *Expression of Src in random co-culture of breast cancer cells (BCCs) and MCF10A*. Upper panel, representative immunoblots of Src detected in BT-474 and 21MT-1 cells co-cultured with MCF10A. Lower panel, densitometry of bands normalized with respective monocultures of BT-474 or 21MT-1. Data are mean ± SD; n=3 independent experiments.

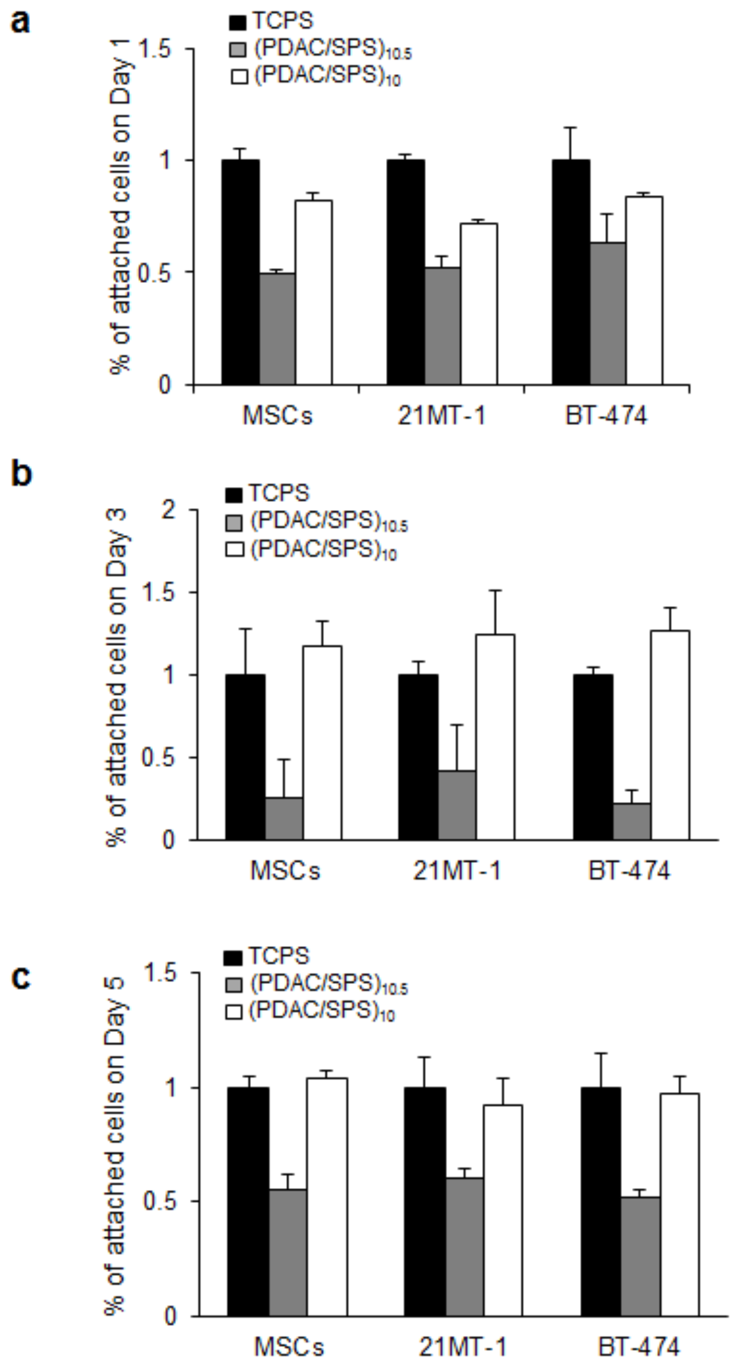

Supplementary Figure 2: *Attachment of BCCs (BT-474 and 21MT-1) and MSCs on fabricated surfaces*. Attachment of cells on $(PDAC/SPS)_{10.5}$ and $(PDAC/SPS)_{10}$ surfaces was determined with MTT on day 1, day 3 and day 5. Cells grown on TCPS were used as control. Mean ± SD, n=3 independent experiments.

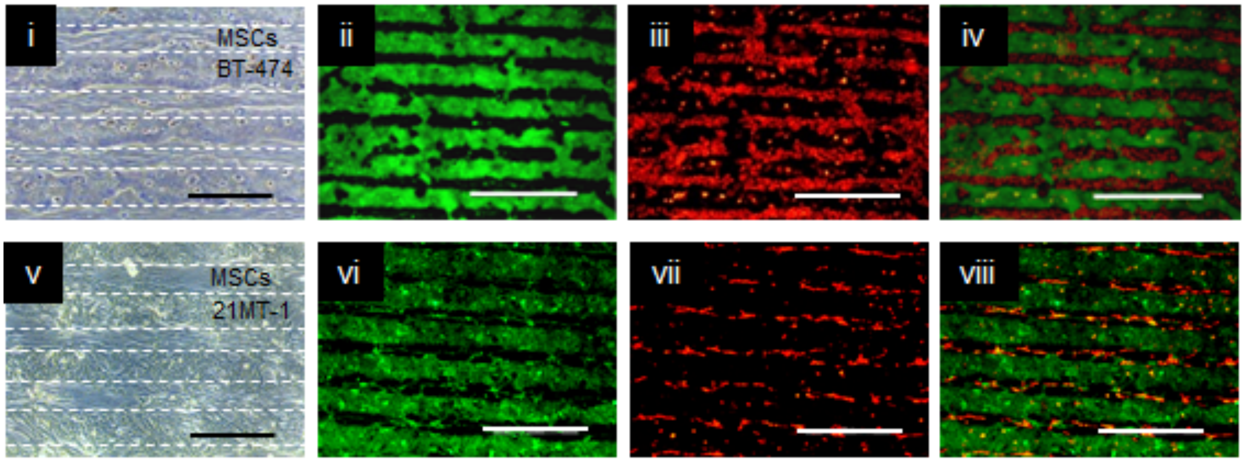

Supplementary Figure 3: *Phase and Fluorescent images of patterned co-culture of BCCs (green) and MSCs (red)*. Upper panel, phase images of BT-474 and MSCs co-culture (i), monochrome images of BT-474 (ii) and MSCs (iii), and merged image shows patterned co-culture of BT-474 and MSCs (iv). Lower panel, phase images of 21MT-1 and MSCs co-culture (v), monochrome images of 21MT-1 (vi) and MSCs (vii), and merged image shows patterned co-culture of 21MT-1 and MSCs (viii). Scale bar 500 μm (ii, iii, iiv, vi, vii, viii) and 200 μm (i, v).

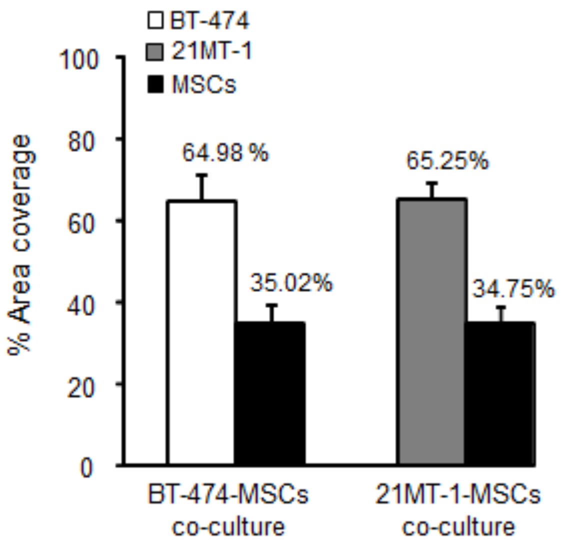

Supplementary Figure 4: *Percentage area coverage of BCCs and MSCs in patterned co-culture.* Area covered by breast cancer cells and MSCs was determined with software ProgRes CaptureProv3.8.0 (Zeiss) provided by Axiovert40 Zeiss inverted microscope.

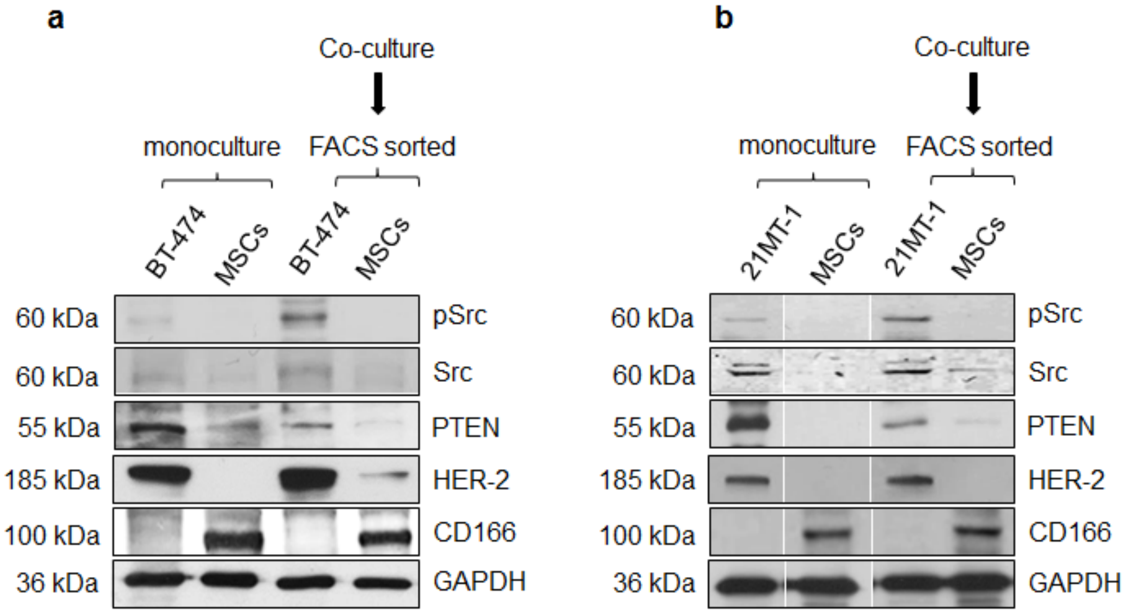

Supplementary Figure 5. *Representative Immunoblots of the indicated proteins from patterned co-culture of BCCs and MSCs after FACS.* (a) Immunoblots show activation of Src and downregulation PTEN in BT-474 cells sorted out from patterned co-culture of BT-474 and MSCs. (b) Immunoblots show activation of Src and downregulation PTEN in 21MT-1 cells sorted out from patterned co-culture of 21MT-1 and MSCs. Efficiency of FACS was determined by probing the blots with breast cancer cells specific marker, HER-2, and MSCs specific marker, CD166. GAPDH was used as loading control.

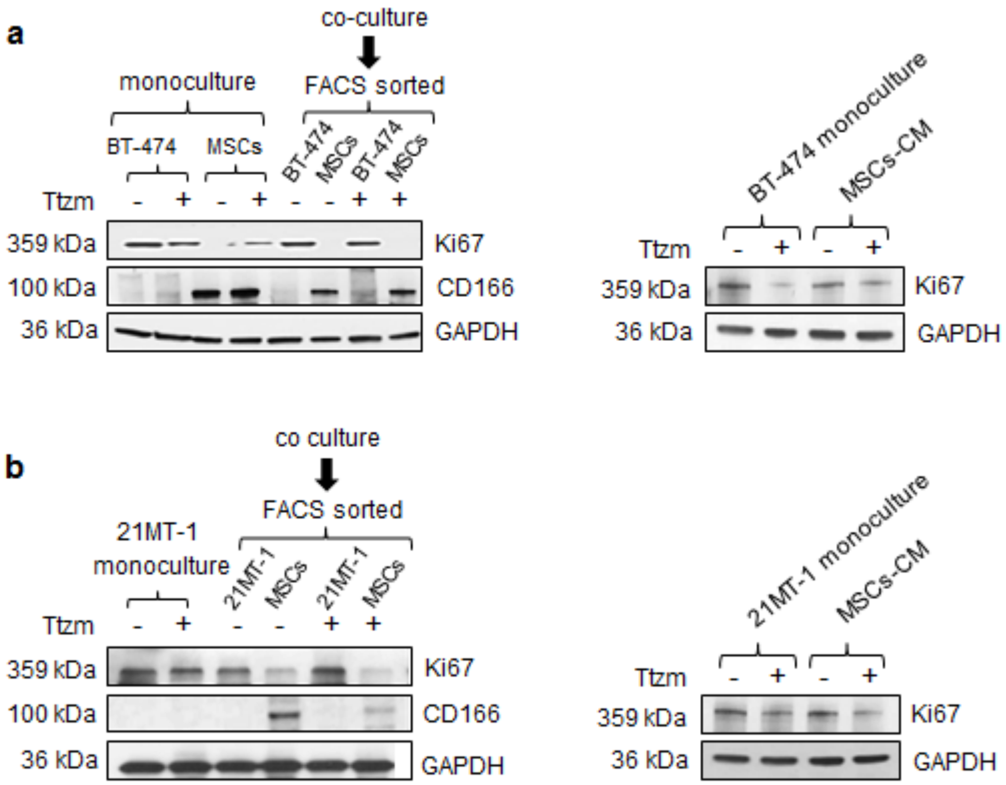

Supplementary Figure 6: *Representative Immunoblots of Ki67 from random co-culture of BCCs and MSCs, and breast cancer cells treated with MSCs-CM.* (a) Left, western blot analysis of Ki67 in BT-474 cells sorted by FACS after co-culture with MSCs in the presence or absence of trastuzumab (Ttzm). Right, BT-474 cells exposed to MSCs-CM in the presence or absence of Ttzm. (b) Left, western blot analysis of Ki67 in 21MT-1 cells sorted by FACS after co-culture with MSCs in the presence or absence of Ttzm. Right, 21MT-1 cells exposed to MSCs-CM in the presence or absence of Ttzm.

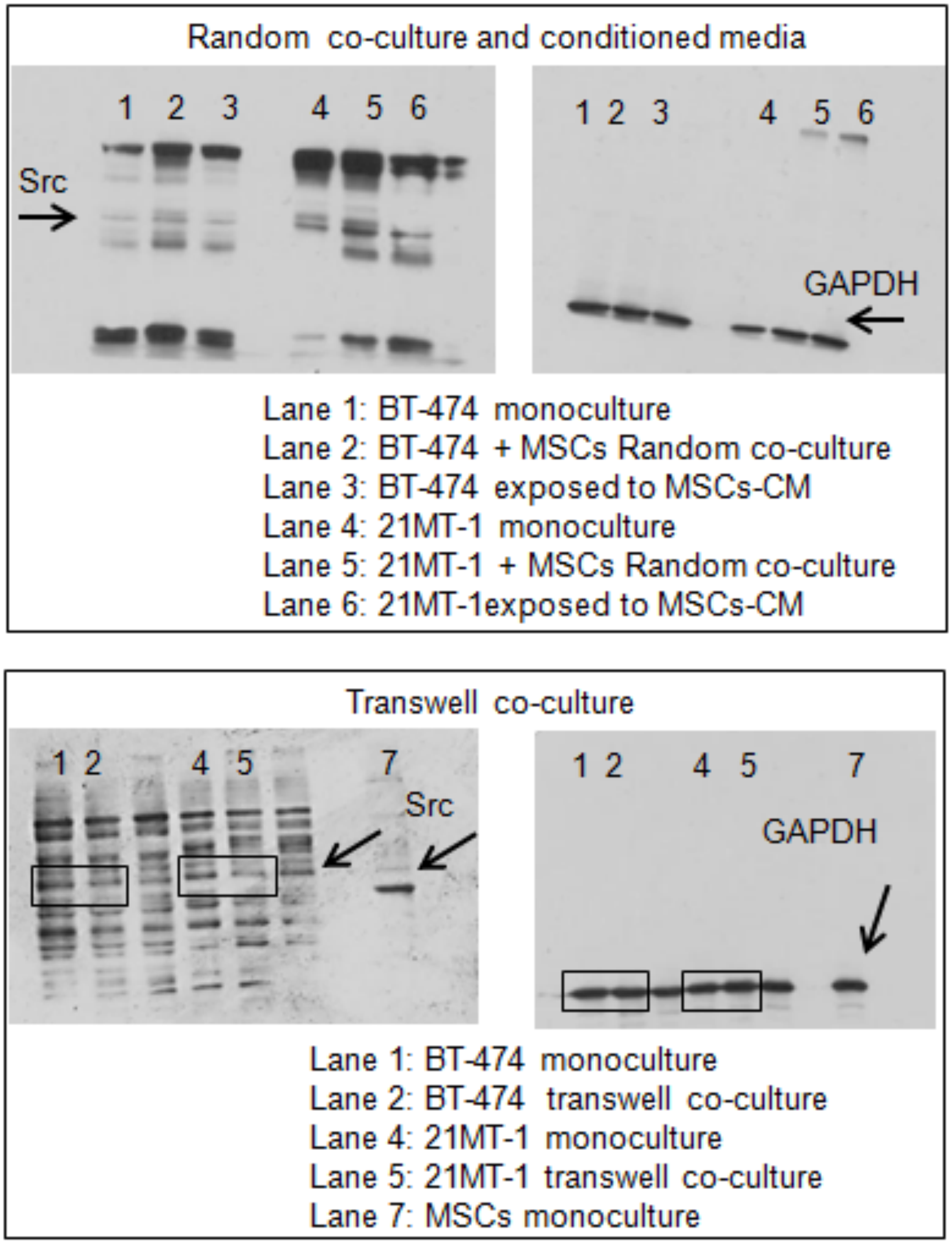

Supplementary Figure 7: Full blots of figure 1a.

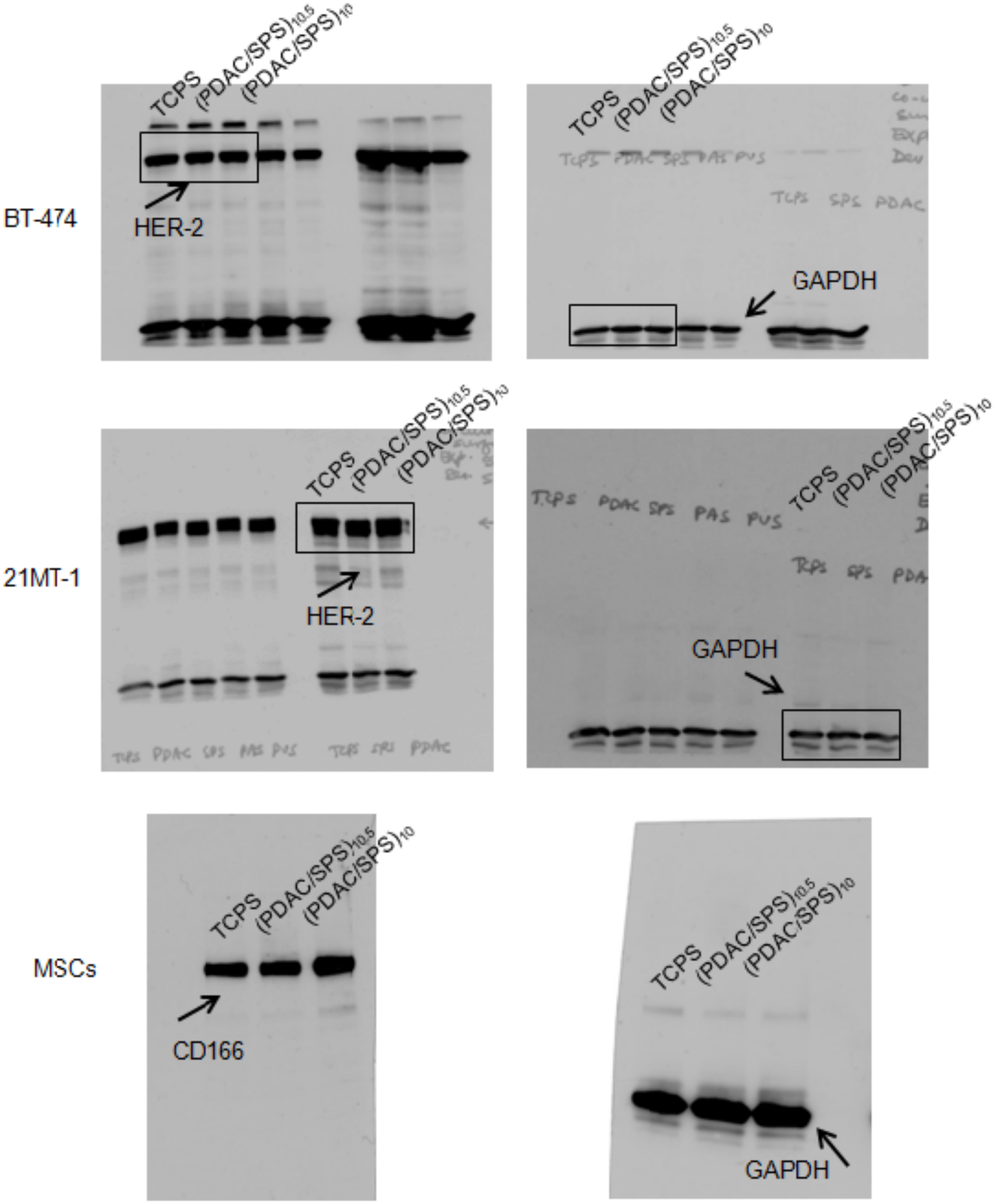

Supplementary Figure 8: Full blots of figure 2b.

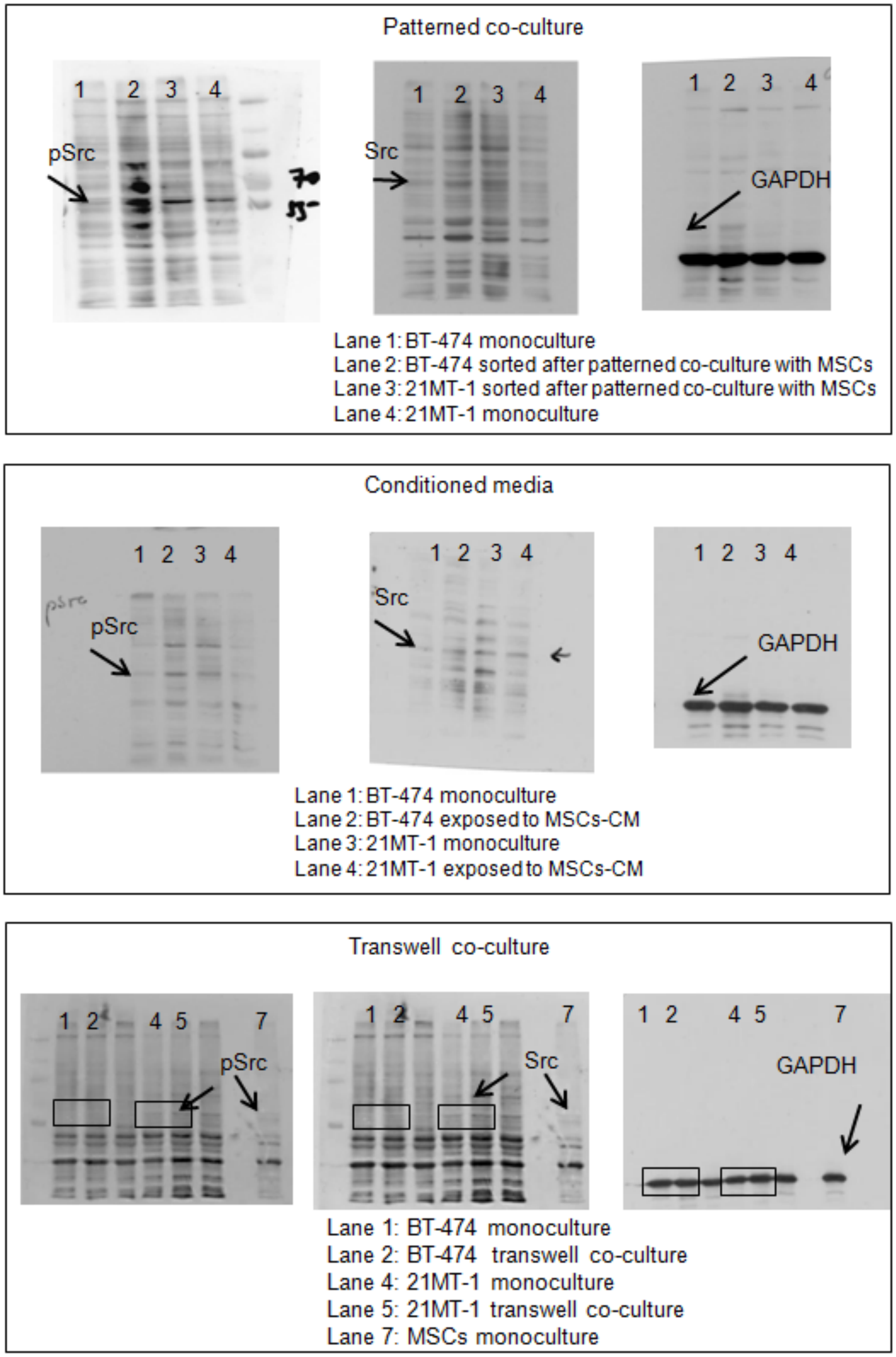

Supplementary Figure 9: Full blots of figure 3b.

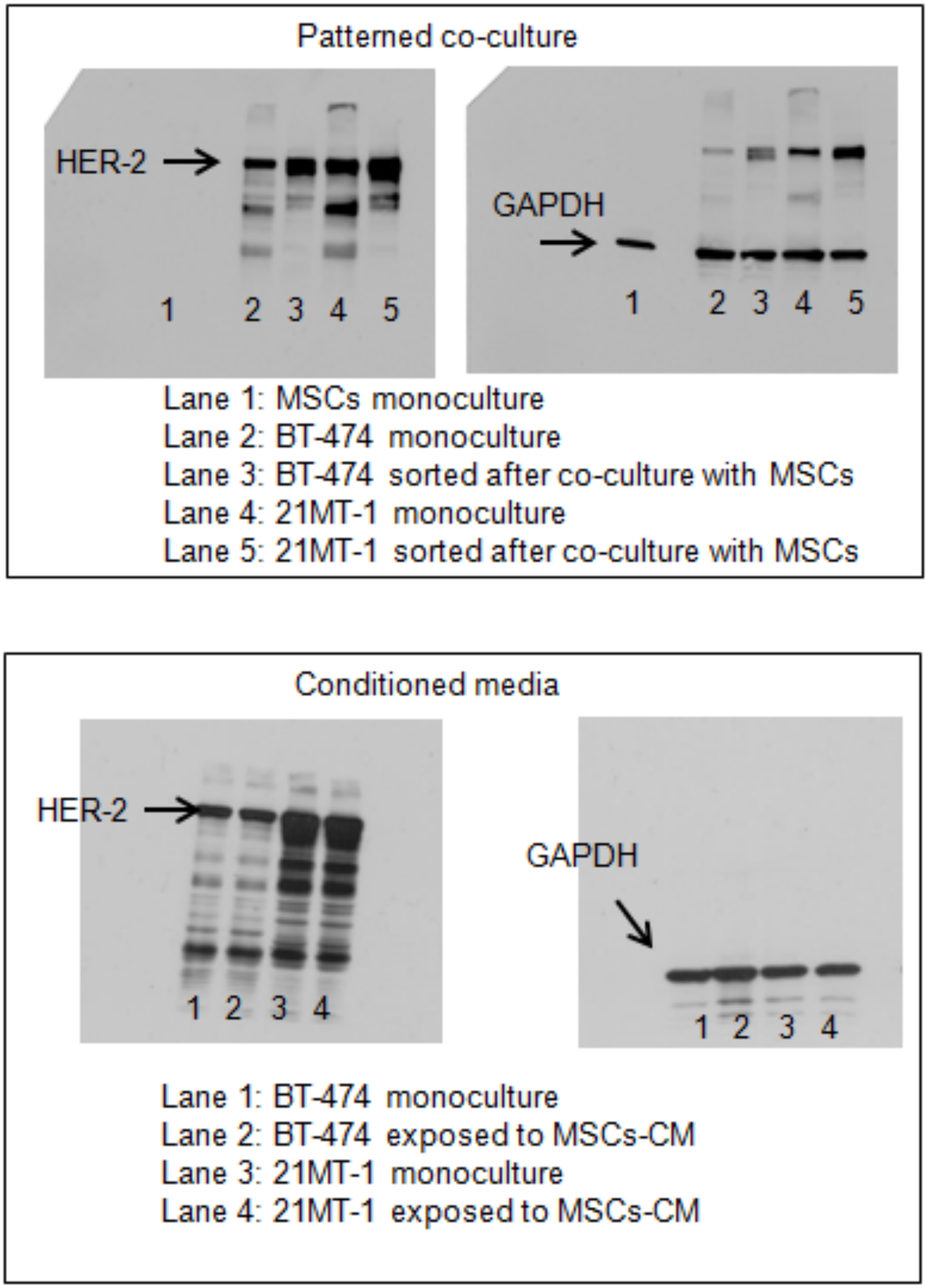

Supplementary Figure 10: Full blots of figure 4b.

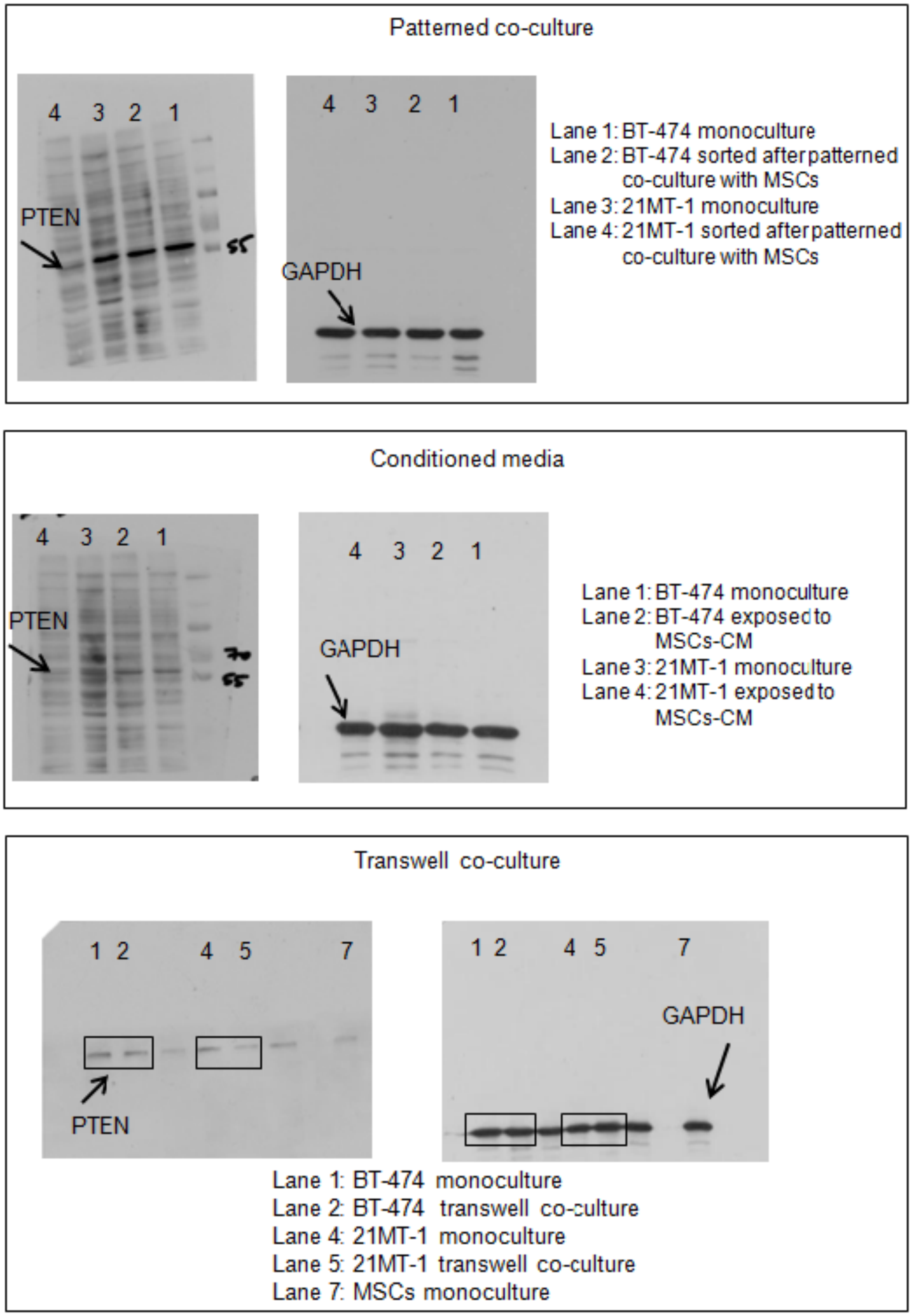

Supplementary Figure 11: Full blots of figure 4c.

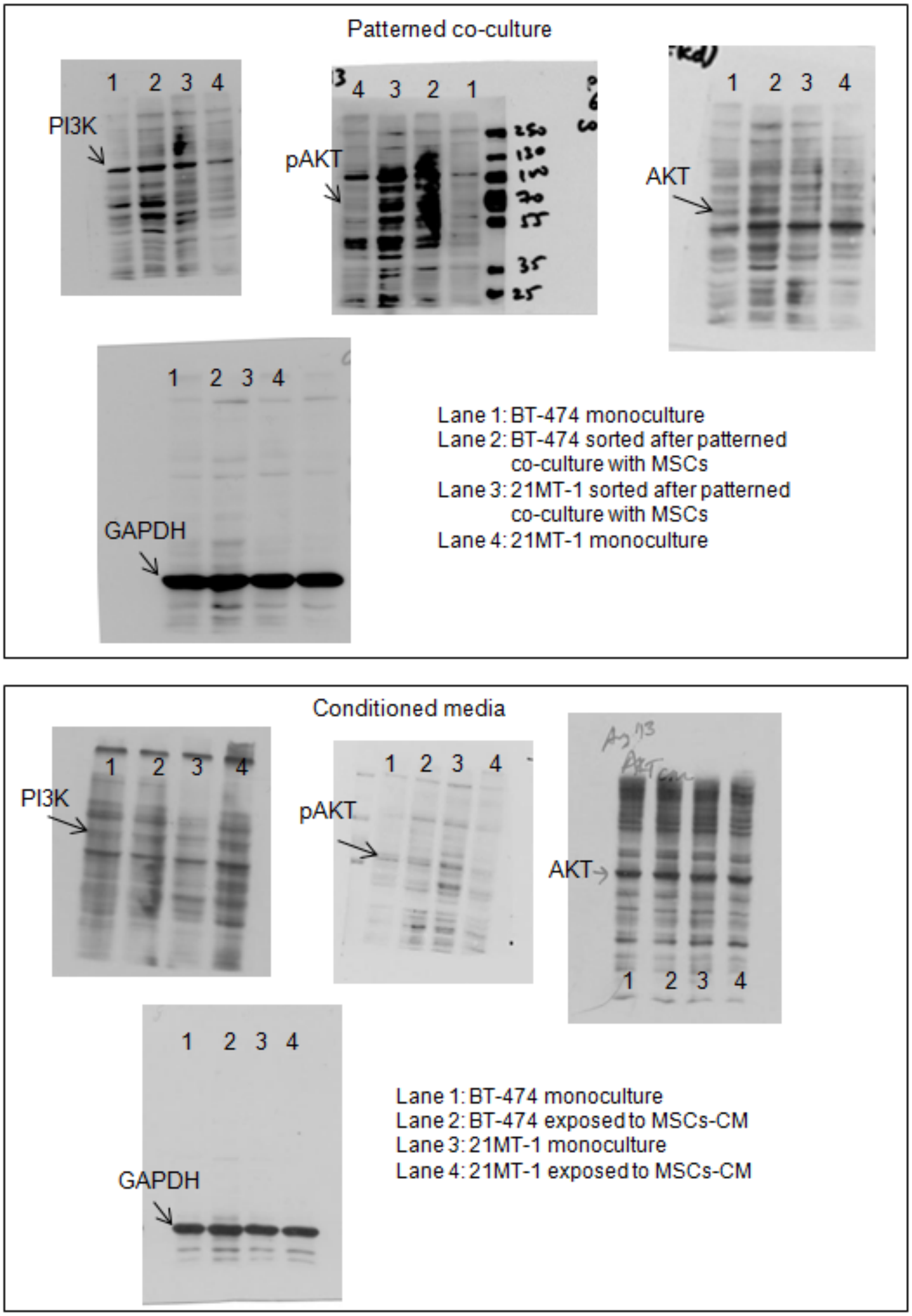

Supplementary Figure 12: Full blots of figure 4d.

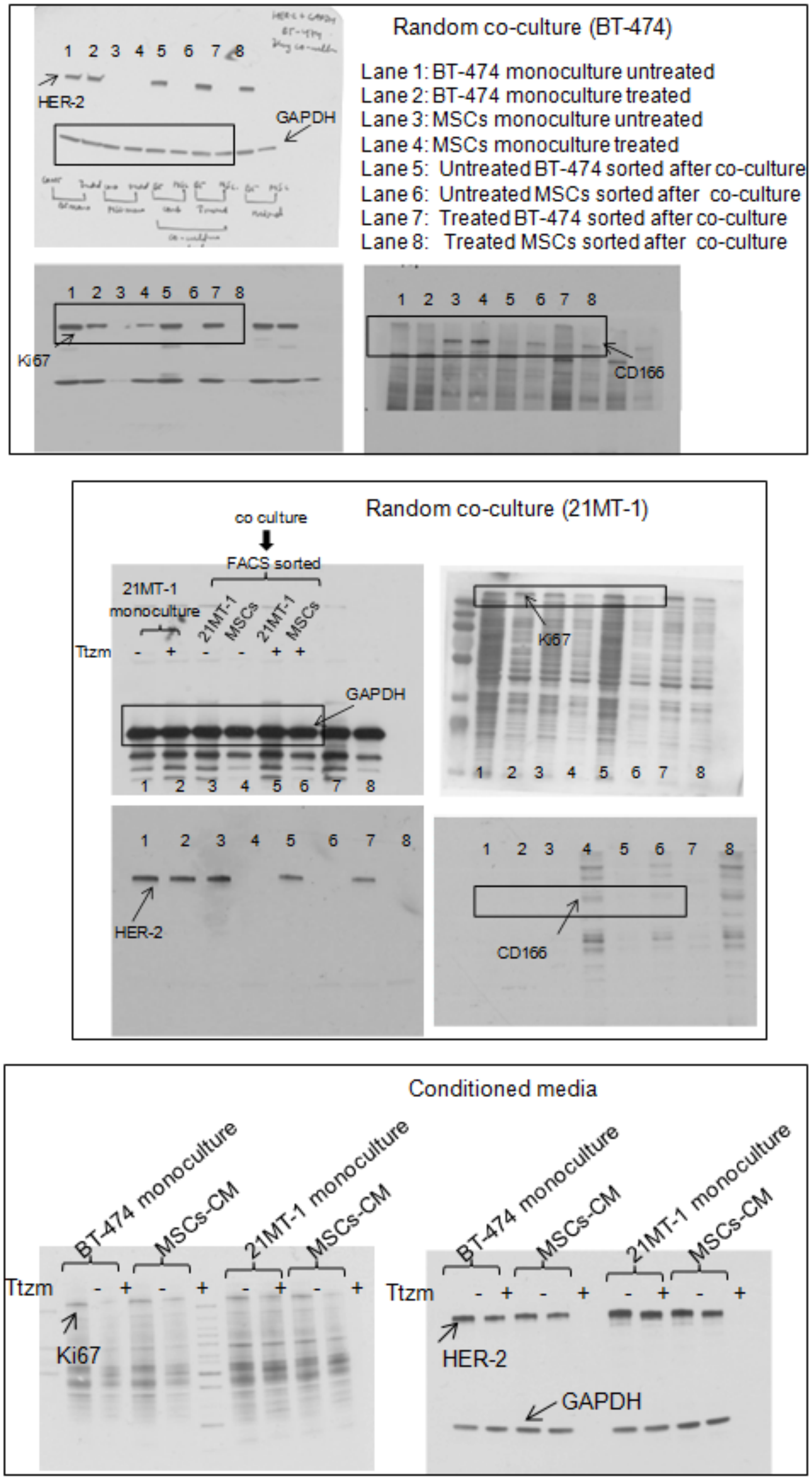

Supplementary Figure 13: Full blots of figure 5b and supplementary figure 6.

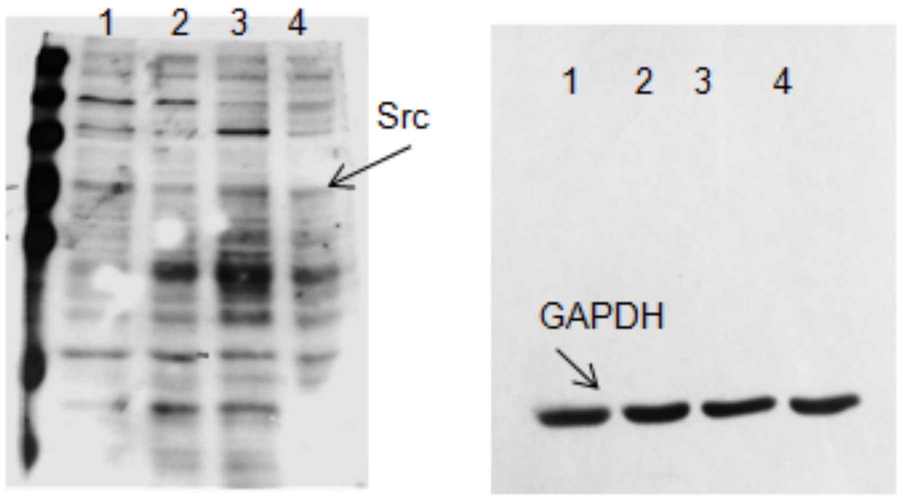

Lane 1: BT-474 monoculture
Lane 2: BT-474 + MCF10A random co-culture
Lane 3: 21MT-1 monoculture
Lane 4: 21MT-1 + MCF10A random co-culture

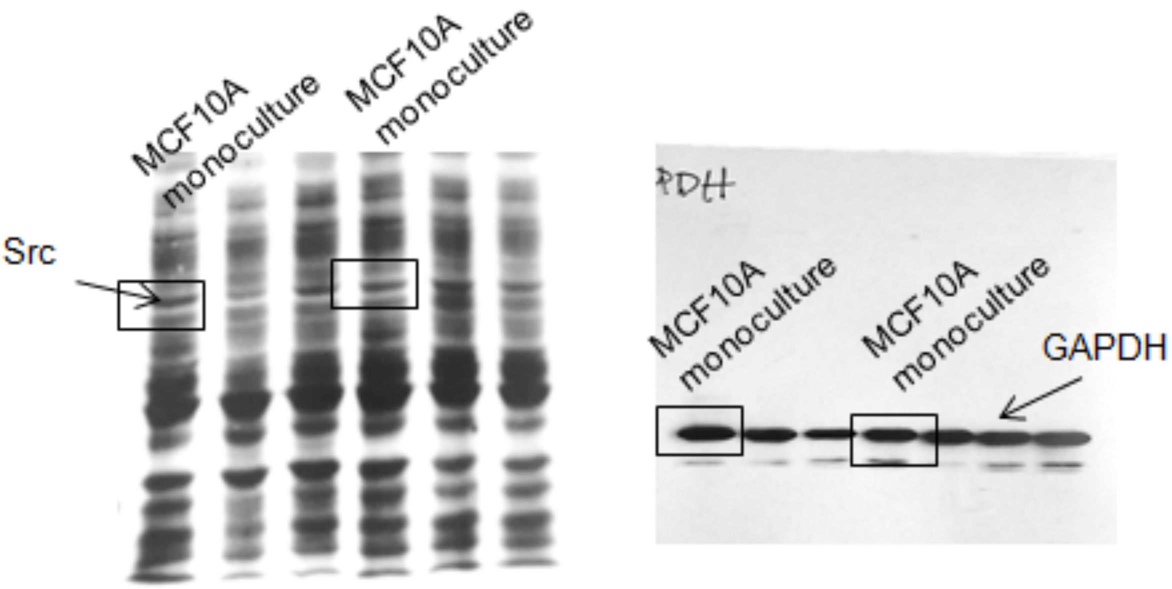

Supplementary Figure 14: Full blots of supplementary figure 1.

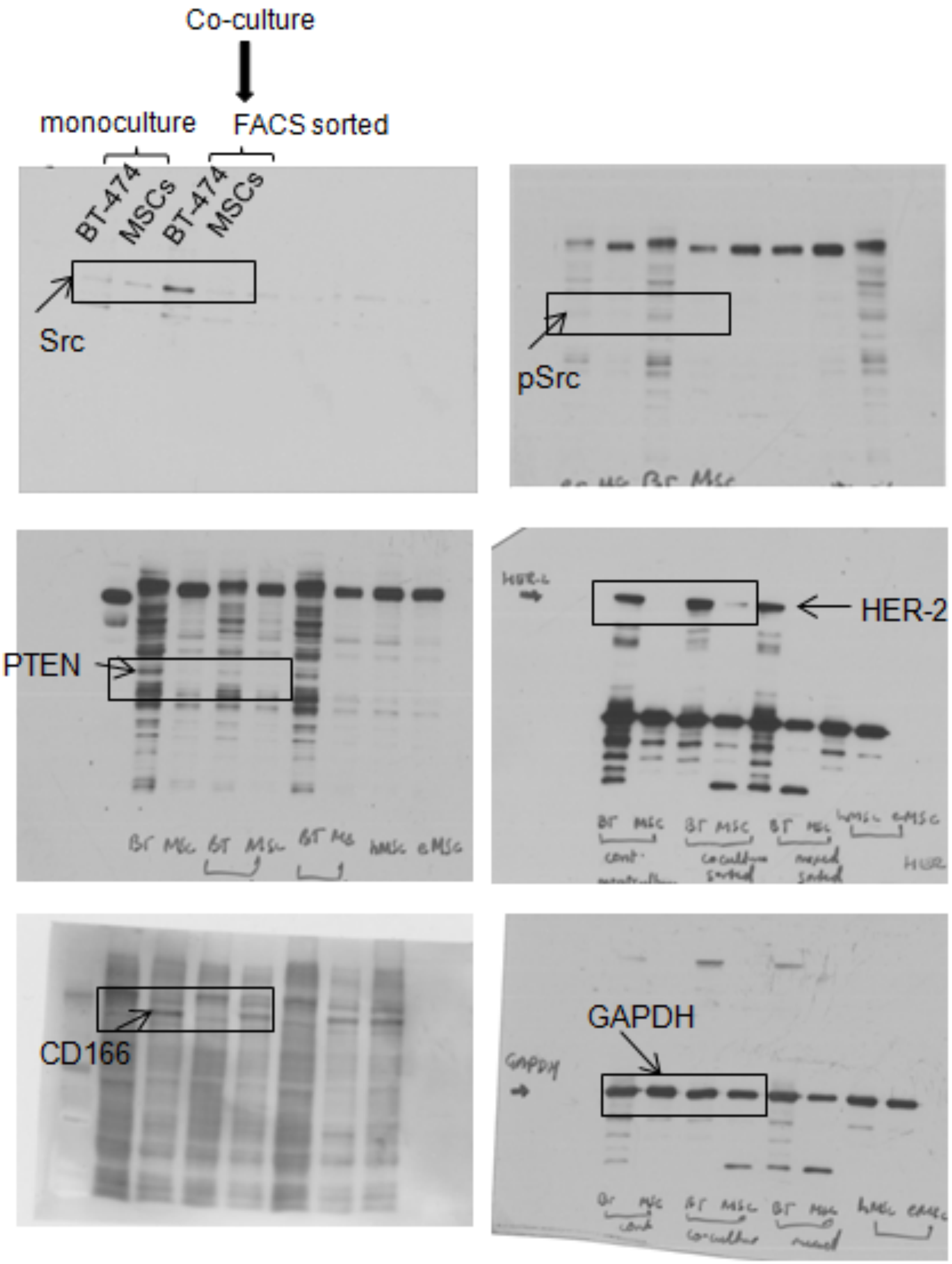

Supplementary Figure 15: Full blots of supplementary figure 5a.

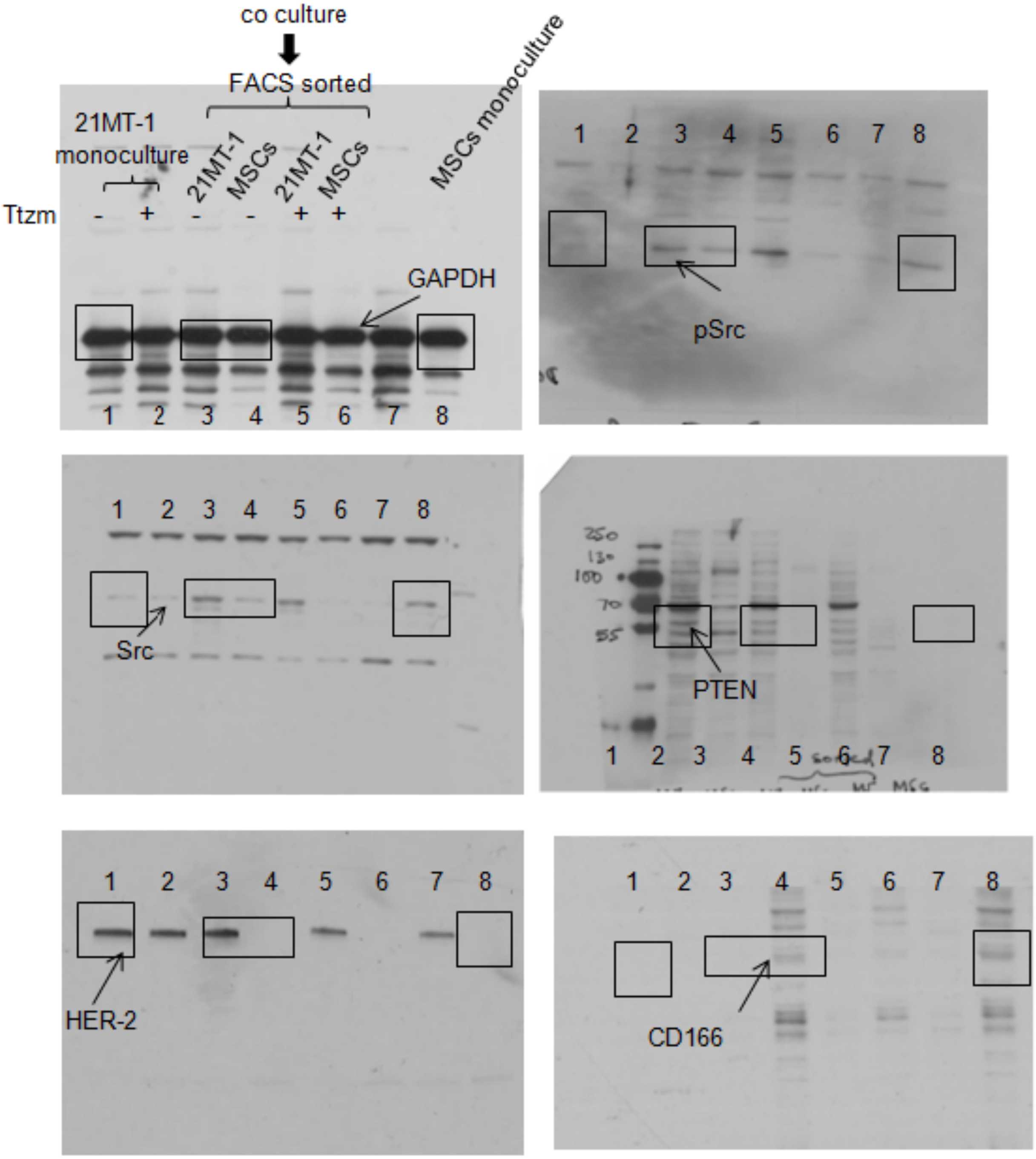

Supplementary Figure 16: Full blots of supplementary figure 5b.

References:

1 Kidambi, S., Lee, I. & Chan, C. Primary Neuron/Astrocyte Co-Culture on Polyelectrolyte Multilayer Films: A Template for Studying Astrocyte-Mediated Oxidative Stress in Neurons. *Advanced functional materials* **18**, 294-301, doi:10.1002/adfm.200601237 (2008).
2 Kidambi, S. *et al.* Patterned co-culture of primary hepatocytes and fibroblasts using polyelectrolyte multilayer templates. *Macromolecular bioscience* **7**, 344-353, doi:10.1002/mabi.200600205 (2007).
3 Kidambi, S., Lee, I. & Chan, C. Controlling primary hepatocyte adhesion and spreading on protein-free polyelectrolyte multilayer films. *Journal of the American Chemical Society* **126**, 16286-16287, doi:10.1021/ja046188u (2004).
4 Daverey, A., Mytty, A. & Kidambi, S. Topography Mediated Regulation of HER-2 Expression in Breast Cancer Cells. *NanoLife* **2** (2012).
